# Using Species Distribution Models to Predict Potential Landscape Restoration Effects on Puma Conservation

**DOI:** 10.1371/journal.pone.0145232

**Published:** 2016-01-06

**Authors:** Cintia Camila Silva Angelieri, Christine Adams-Hosking, Katia Maria Paschoaletto Micchi de Barros Ferraz, Marcelo Pereira de Souza, Clive Alexander McAlpine

**Affiliations:** 1 University of São Paulo, Water Resources and Environmental Studies Centre, São Carlos School of Engineering, São Carlos, SP, Brazil; 2 University of Queensland, School of Geography, Planning and Environmental Management, Brisbane, QLD, Australia; 3 University of São Paulo, Forest Science Department, Luiz de Queiroz College of Agriculture, Piracicaba, SP, Brazil; 4 University of São Paulo, Biology Department, Ribeirão Preto School of Philosophy, Science and Literature, Ribeirão Preto, SP, Brazil; University of Porto, PORTUGAL

## Abstract

A mosaic of intact native and human-modified vegetation use can provide important habitat for top predators such as the puma (*Puma concolor*), avoiding negative effects on other species and ecological processes due to cascade trophic interactions. This study investigates the effects of restoration scenarios on the puma’s habitat suitability in the most developed Brazilian region (São Paulo State). Species Distribution Models incorporating restoration scenarios were developed using the species’ occurrence information to (1) map habitat suitability of pumas in São Paulo State, Southeast, Brazil; (2) test the relative contribution of environmental variables ecologically relevant to the species habitat suitability and (3) project the predicted habitat suitability to future native vegetation restoration scenarios. The Maximum Entropy algorithm was used (Test AUC of 0.84 ± 0.0228) based on seven environmental non-correlated variables and non-autocorrelated presence-only records (n = 342). The percentage of native vegetation (positive influence), elevation (positive influence) and density of roads (negative influence) were considered the most important environmental variables to the model. Model projections to restoration scenarios reflected the high positive relationship between pumas and native vegetation. These projections identified new high suitability areas for pumas (probability of presence >0.5) in highly deforested regions. High suitability areas were increased from 5.3% to 8.5% of the total State extension when the landscapes were restored for ≥ the minimum native vegetation cover rule (20%) established by the Brazilian Forest Code in private lands. This study highlights the importance of a landscape planning approach to improve the conservation outlook for pumas and other species, including not only the establishment and management of protected areas, but also the habitat restoration on private lands. Importantly, the results may inform environmental policies and land use planning in São Paulo State, Brazil.

## Introduction

Humans now dominate the earth, negatively impacting on the survival of many wildlife species [1]. However, a variety of habitat types in human-modified landscapes can be used by wildlife, with the pattern of utilization varying according to species’ life-histories [2]. These human-modified landscapes are heterogeneous mosaics of land uses such as agricultural lands (croplands and pastures), urban areas, roads, watercourses and remnant patches of native vegetation [3] which can be managed for the conservation of biodiversity, even if most of the native vegetation has been converted to human land uses [4]. Adopting a landscape-scale approach is now recognized as vital to conserving wildlife outside protected areas; it is especially relevant to the conservation of large carnivore populations which require large territories for movement and foraging [5].

Large carnivores are particularly sensitive to habitat loss and fragmentation [6]. However, some species utilize the land use matrix such as agricultural crops for hunting and dispersal movements. For example, puma (*Puma concolor*), gray wolf (*Canis lupus lupus*) and maned wolf (*Chrysocyon brachyurus*) have been found in many different types of land-use around the world, such as pasture, croplands and even urban areas [6], [7], [8]. This coexistence of large carnivores and humans causes conflicts, such as retaliatory killing by humans due to livestock predation, for example, of lynx (*Lynx linx*) [9], wolf (*Canis lupus*) [10], lion (*Panthera leo*), leopard (*Panthera pardus*) and spotted hyena (*Crocuta crocuta*) [11], puma and jaguar (*Panthera onca*) [12], [13]. It is crucial, therefore, to incorporate human-modified landscapes into a new conservation paradigm to resolve conflicts between people and large predators around the globe [14].

In the Neotropics, the jaguar and the puma (originally coexisted across the entire jaguar range, but now jaguars are officially extinct in El Salvador, Uruguai [15] and in the United States (some individuals occasionally cross from Mexico into the southern United States) [16]. In Brazil, they have practically disappeared from the northeast, south and the highly-fragmented Atlantic forest regions [17]. A high extinction risk of the jaguar has recently been reported in the highly-fragmented Atlantic rainforest biome [18], [19]. Jaguars are already locally extinct in many areas of São Paulo State, Brazil. However, the puma still survives in this threatened region and is now the only top predator. This species has been considered a trigger for a trophic cascade effect [20], with population and geographic range declines resulting in significant cascading trophic interactions and substantial effects on other species and ecological processes [21]. With this in mind, effective recovery strategies for predators such as the puma may be pivotal to improving biodiversity conservation in general [22].

The puma can be found in different types of land cover with various levels of human modification (e.g., [23], [24], [25], [26], [27]). However, the species is believed to avoid highly-modified areas [28] and to prefer habitats containing proportionally more native vegetation cover [24]. It remains unclear whether avoidance of highly-modified habitats is direct effect of poor habitat quality or the result of prey avoiding human activity. For example, Sweanor et al. [29] suggest that a landscape containing more native vegetation and less human activities must be a better scenario for puma conservation. Strategies to restore native vegetation at the landscape-scale may improve the conservation outlook for puma.

The puma is well documented in North America, but there is a lack of studies to provide information about the genetics and ecology of pumas in South America, especially in Brazil. It is argued that the species have only recently recolonized south and southeast regions, following decrease in logging activities after depletion of the Atlantic rainforest in the 1960s [27]. Although there are evidences that puma may act as source–sink metapopulation structure within these areas (e.g. [25], [30]), dispersing cougars do not necessarily follow the path of least resistance (e.g. [31]). The first estimate of the abundance of pumas in a human-dominated landscape in São Paulo State was published last year [32].

This study aims to explore the effects of restoration scenarios on the puma’s habitat suitability in human-modified landscapes. To achieve this, species distribution models (SDMs) incorporating restoration scenarios were developed using the specie’s occurrence information to (1) map habitat suitability of pumas in São Paulo State, Southeast, Brazil; (2) test the relative contribution of environmental variables ecologically relevant to the species habitat suitability and (3) project the predicted habitat suitability onto future native vegetation restoration scenarios.

Mapping habitat suitability for species using species distribution models have been increasingly applied as conservation planning tools for predicting impacts of climate change and land use changes on biodiversity (e.g. [33], [34], [35], [36]). These models can provide insights into systematic conservation planning, informing the decision making process [37]. São Paulo State was considered an appropriate study area to conduct this study because it is the most populous state in Brazil [38] and large areas of highly fragmented native vegetation [39].

## Methods

### Study area

**Fig 1:**
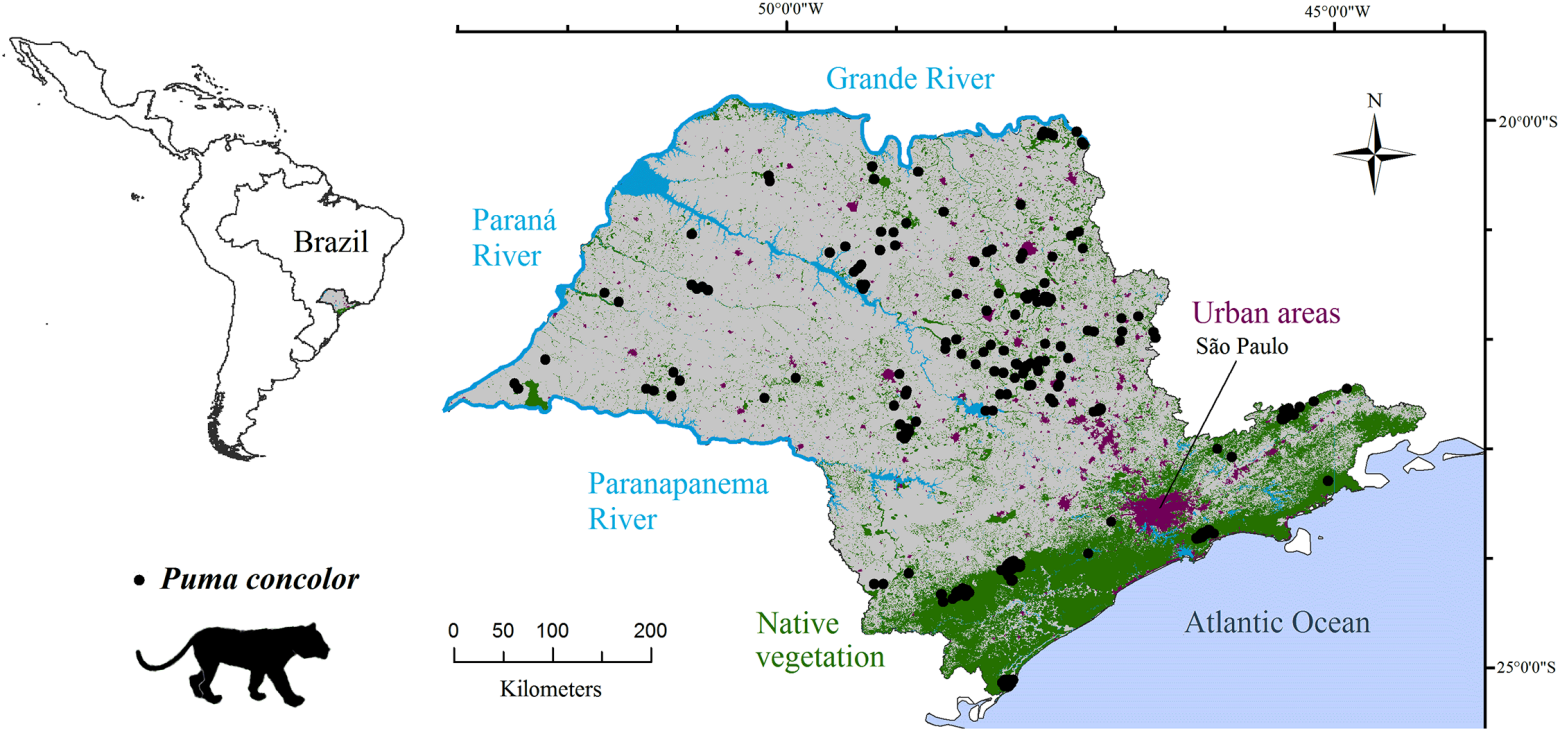
Study area map. Land use and pumas’ occurrence records (2001–2012) in São Paulo State, Southeast, Brazil. This figure was elaborated by the first author using software ArcGIS 10.1 and IrfanView 4.37.

The study incorporated the entire territory of São Paulo State, Southeast, Brazil (248 209 km^2^). It is the most developed state in Brazil with a human population estimated at approximately 43.65 million inhabitants in 2013 and varying from 6 persons /km^2^ in small towns to 7 398 persons/km^2^ in the capital São Paulo [38]. During the 20^th^ century, São Paulo State experienced rapid land use changes due to human population growth and agricultural development [40]. During the last decade, many cattle pastures have been converted to sugar cane plantations [41]. São Paulo State is covered by two Atlantic Forest sub-regions (Serra do Mar and Interior). Approximately 4.7 million ha of native vegetation patches remain, with over 2.94 million ha (62.5%) clustered in a 100 km buffer inland from the Atlantic Ocean Coast (calculated in ArcGIS 10.1 based on Sparovek et al. [42]). São Paulo State is delimited by the Atlantic Ocean (Southeast boundary), and three main rivers: Paranapanema River (Southwest boundary), Paraná River (West boundary) and Grande River (North boundary) (Fig 1). These rivers were not tested as effective barriers to the puma dispersion in the region, although large watercourses were considered by experts as effective barriers to puma dispersal in Southwest United States [43].

A few studies have found that average home range size in the study area varies from 51 to 140 km^2^ in the São Paulo North East region [44] and 114 km^2^ in São Paulo Central East region [45] with density varying from 0.30 to 0.74/100 km² in the East Coast [46]. Importantly, pumas have been observed in mosaics of native vegetation and exotic forest plantations dominated by Pinus and Eucalyptus spp. in this area [47]. Those areas usually are under certified timber management.

### Species occurrence records

Presence-only puma records for the period 2001–2012 (n = 495) in São Paulo State were downloaded from the Species Link network [48] and provided by the Puma Conservation National Act Plan research collaborators. All data used (including from Species Link network) were collected and provided by specialists or government staffs (e.g. IBAMA, ICMBio). We avoid records derived from museum and those ones which the data provider could not be easily identified to support data accuracy. Duplicate records were removed in ArcGIS *10*.*1*, which left 343 spatially independent records for the analysis (S1 Table). Instead of reducing the effect of sampling bias by spatially filtering the occurrence dataset, we develop a sampling probability surface called bias grid to reduce sampling bias in geographic space when some areas in the landscape are sampled more intensively than others [49]. We followed Clements et al. [50] to develop a bias grid weighted by a Gaussian Kernel with a standard deviation (SD) based on the estimated puma’s dispersal distance divided by the weighted number of terrestrial cells in the neighborhood [51], thereby avoiding the effect of the coastline. A Gaussian function was applied using ArcGIS 10.1: Exp (— ([d] ^ 2) / (2 * SD ^ 2) ) where d = the distance between each 10,000 background points and presence points measured in GME tool version 0.7.2.1 RC2 [52], and standard deviation (SD) = maximum dispersal distance of pumas described by Maehr et al. as 68.4 km [53]. The final values were interpolated using the Natural Neighbor algorithm to create a smooth surface from points and converted to ASCII format (S1 Fig).

### Environmental variables

The ecological relationships considered in the literature to be relevant for pumas were considered as potential explanatory continuous variables (n = 13) to guide the species distribution modelling (SDM) (S2 Table). The variables were: (1) percentage of native vegetation, (2) distance to native vegetation, (3) edge density of native vegetation patches, (4) percentage of exotic forest crops, (5) distance to exotic forest crops, (6) distance to protected areas,(7) watercourse density, (8) distance to watercourses, (9) distance to roads, (10) road density, (11) elevation and (12) slope and (13) distance to urban areas.

A Pearson’s correlation analysis was applied to test the independence between each pair of environmental variables using the package Hmisc 3.10 in R 2.15.1 [54]. Variables were excluded from the analysis if their correlation index was ≥ ± 0.5 (S3 Table), following Booth et al. [55]. These authors suggested that, if a pair of variables has a correlation coefficient ≥ ± 0.5, then they should be considered proxies of one another, and one of the variables should be removed. The variables showing the highest gain to the models in preliminary Maxent runs were retained from a group of correlated variables.

The following environmental variables were subsequently selected as predictors: percentage of native vegetation, percentage of exotic forest crops, edge density, watercourse density, road density, elevation, and slope. Distance to protected areas, distance to native vegetation and distance to roads were highly correlated with percentage of native vegetation and they were therefore excluded from the analysis. Distance to urban areas and distance to roads were also excluded due to their high correlation with road density. Distance to watercourses was excluded because it was correlated with density of watercourses.

All environmental variables selected (Table 1) were extracted by the mask of São Paulo State, projected to South American Datum 1969 Albers Equal-Area Conic Projection spatial reference defined by custom to minimize area distortion and resampled to the largest cell size of all input datasets (~90 x 90 meters) in ArcGIS 10.1. They were then converted to ASCII grid format for the analysis.

**Table 1:** Environmental variables used to develop the models for pumas in São Paulo State, Brazil (See S4 Table for the environmental variables not used to develop the models).

Environmental variable	Description and source
**Percentage of native vegetation**	Geotiff binary map (values 0 or 1) of natural vegetation in Brazil*. Sum of natural vegetation cells (value 1) divided by the total number of cells with respect to the upper-left corner of a 10 x 10 km rectangle neighborhood* using Focal Statistics and multiplied by 100 (%).
**Percentage of exotic forest crops**	Geotiff binary map (values 0 or 1) of eucalyptus and/or pine plantations in Brazil. Sum of cells (value 1) divided by the total number of cells with respect to the upper-left corner of a 10 x 10 km rectangle neighborhood** using Focal Statistics and multiplied by 100 (%).
**Edge density of vegetation**	Geotiff binary map (values 0 or 1) of natural vegetation in Brazil* converted to a polygonal feature to performing area and perimeter calculations on attribute table. Perimeter divided by area using Python Field Calculator. Final values converted into raster image (m/m^2^).
**Elevation**	Geotiff continuous map of digital elevation model of São Paulo State map originally in the South American Datum 1969***. Raster continuous dataset projected to Albers Equal-Area Conic Projection using Data Management tool Project Raster (bilinear resampling technique) (m).
**Slope**	Slope percentage rise calculated from Elevation map in Albers Equal-Area Conic (meters) using Spatial analyst tool Surface (%).
**Road density**	Kernel density of roads in São Paulo State**** with respect to the length per square map unit in ~ 100 km^2^ neighborhood** (m/m^2^).
**Watercourse density**	Kernel density of water bodies in São Paulo State with respect to the length per square map unit in ~ 100 km^2^ neighborhood** (m/m^2^).

*Land use binary maps (urban areas, native vegetation and exotic crops) originally in World Geodetic System 1984 (cell size 0.00077 x 0.00083) developed by Sparovek et al. [37].

**Density and connectivity variables were based on the puma’s average home range of 100 km^2^ as found within the study region [44], [45].

*** Elevation and Slope continuous maps originally in South American Datum 1969 (cell size 0.00083 x 0.00083) developed by Weber et al. based on the Shuttle Radar Topography Mission*—*SRTM [56]

**** Road density was based on state and federal highways. It does not include unpaved roads.

### Restoration Scenarios

The restoration scenarios were created based on the land use binary maps (urban areas, native vegetation and exotic crops) of São Paulo State [42]. Firstly, areas with ≥ 70% percentage of agriculture lands were selected (based on the sum of natural vegetation cells divided by the total number of cells with respect to the upper-left corner of a 10 x 10 km rectangle neighborhood using Focal Statistics and multiplied by 100) to represent areas that potentially could be restored to native vegetation. Secondly, areas with native vegetation percentage values of ≤ 10%, ≤ 20%, ≤ 30% were selected as areas with restoration needs. Finally, these areas of potential restoration and restoration needs were overlapped.

Three scenarios were then created simulating native vegetation increases across the landscape. These represented steps in a future restoration process, when native vegetation would be restored by 10%, 20% and 30%. Changes of percentage of native vegetation did not influence changes in any other variables used in the modelling process.

The values of the restoration scenarios were selected based on ecological assumptions reflecting the plasticty of the puma to use landscapes with moderate levels of anthropogenic modification [57]. Specifically, landscapes with less than 20–30% of forest cover tend to hold depleted faunal communities in the Brazilian Atlantic Forest [58], [59], [60], thereby negatively affecting the foraging ecology of the species. Furthermore, studies suggest extinction thresholds are observed below 30% of remaining habitat [61] and the distance among patches increases exponentially around 10–20% of remaining habitat [62]. In addition to these ecological assumptions, the minimum native vegetation cover rule in private lands established by the Brazilian Forest Code is 20% in the study region [63]. A similar modelling process and scenarios analysis based on these code specifications was applied by Ferraz et al. [64].

### Model development

The Maximum Entropy Software (Maxent v. 3.3.3 k) [65], was applied to develop a species distribution model for pumas and to make projections for future restoration scenarios. Maxent has shown a better perform than other presence-only data modelling methods (e.g. GARP) due to its ability to fit complex responses and to select a relevant set of variables [66]. The Maxent algorithm uses the environmental variables that are ecologically relevant to the species from the presence-only data and a background sample (a random sample of available environmental conditions) to calculate a habitat suitability index, indicating where the species is most likely to occur [67]. The logistic output format estimated by Maxent (continuous values from 0 to 1) can be interpreted as the probability that the species was present, conditional on the environmental conditions, but it does not correspond to an explicit model of species occurrence [68] [69]. Refer to Elith and Leathwick [70] for further details on SDMs and Elith et al. [71] for further details on the Maxent algorithm.

Maxent performs well even for modelling incomplete data, limited sample size and biased data [67], [68], [69], which are common problems in studies involving large carnivores. Maxent outperformed other algorithms (ENFA and SVM) in predicting large carnivore distribution [72]. This algorithm has been recently applied to model large carnivores, such as jaguars [73], [64], [17], [19], pumas [74], lions and leopards [72].

Although Maxent is particularly easy to use, it requires that users make informed decisions when preparing data, choosing settings and interpreting outputs [71]. In this study, the SDM was generated by bootstrapping (n = 10) using 70% of the dataset for training and 30% for testing models [75]. Maxent’s default setting sampled 10,000 background points from the study area and set the maximum number of iterations to 500 and the regularization multiplier to one.

The area under the receiver operating characteristic curve (AUC) was used to evaluate the discriminative ability of Maxent models. AUC values greater than 0.75 (a no better than random AUC = 0.5) were considered able to distinguish between presences and potentially unsampled locations [76]. A Jacknife test was applied to measure relative importance of each environmental variable. Maxent measured this by “gain”, which represented how much better the distribution fitted the sample points than the uniform distribution did (uniform distribution gain = 0). The average sample likelihood was exp (x) times higher than that of a random background pixel if Maxent’s gain was x.

Maxent estimated the environmental variables’ contributions to the SDM by “percent contribution” and “permutation importance” values (from 0 to 100%). For the first estimate, the increase in regularized gain was added by Maxent to the contribution of the corresponding variable or subtracted from it if the change to the absolute value of lambda was negative in each iteration of the training algorithm. For the second estimate, the values of that variable on training presence and background data were randomly permuted for each environmental variable in turn. The model was reevaluated on the permuted data, and the resulting drop in training AUC was normalized to percentages. Those values are only heuristically defined as dependent on the particular path that the Maxent code used to get to the optimal solution.

Maxent’s distribution (original) was ‘clamped’ at constant probabilities and projected into novel environments (in this case, restoration scenarios), extrapolating the current conditions to novel combinations of environmental variables [51]. The option ‘projections layer’ file/directory was assigned containing the same environmental variables but a different target percentage of vegetation for each restoration scenario (percentage of native vegetation value ≤ 10%, ≤ 20%, ≤ 30%).

### Zonal Statistics

Threshold-dependent evaluations were avoided in this study because it has been challenged as unnecessary for conservation planning purposes [77]. Maxent’s average distribution model (i.e. probability of presence values) was categorized into three frequency distribution classes (i.e. low 0.00–0.17, medium 0.17–0.31, medium-high 0.31–0.50 and high 0.50–0.99) by Jenks' natural breaks classification method using ArcGIS 10.1 Spatial Analyst tool Reclassify. Jenks' classification is a data clustering method well suited for grouping data with large variances and seeks to reduce variance within groups and maximize variance between groups [78].

A cross-tabulated analysis was used to explore how the landscape habitat suitability changes when native vegetation increases in scenarios because just one variable was changed (i.e. percentage of vegetation). Geotiff binary maps of land cover classes (i.e. native vegetation, exotic forest crops, urban areas and agricultural land) were overlayed to a single raster dataset, creating a land cover map [42]. Fully protected areas in Brazil (municipal, state and national) were combined into a single output polygon feature [79]. A 10 km buffer distance was calculated to accommodate possible analytical edge effects for pumas [43] and the transition zone between protected areas and the surrounding landscape. A 10 km buffer from fully protected areas and also sustainable reserves were combined into a single output polygon feature. The ArcGIS 10.1 Spatial Analyst tool was applied to tabulate the probability of presence areas with land use and protected areas.

## Results

**Fig 2:**
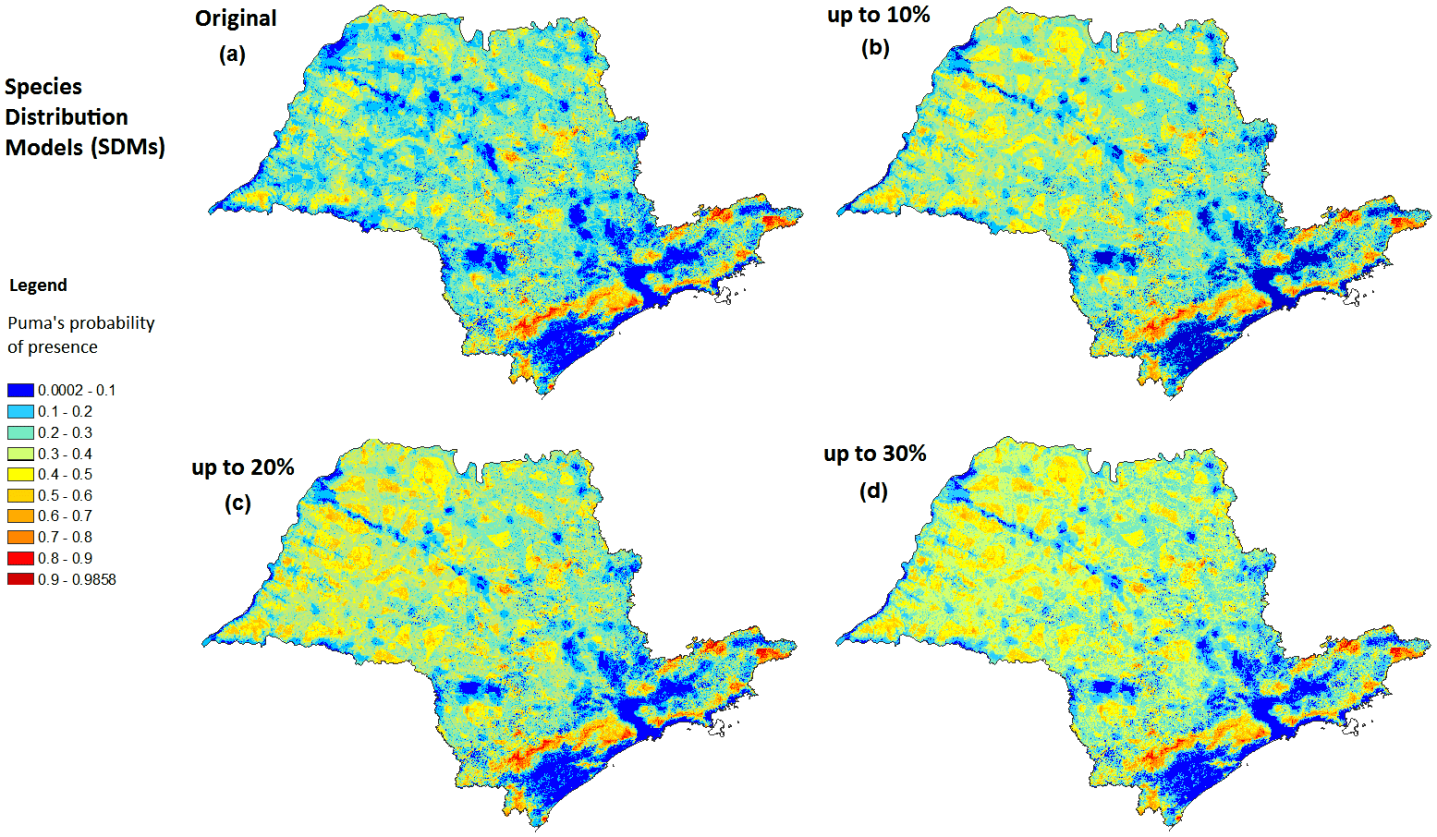
The puma habitat suitability in São Paulo State, Brazil and its projection in three restoration scenarios: (a) original Maxent distribution model average, (b) ≥10% percentage of native vegetation restoration scenario, (c) ≥20% percentage of native vegetation restoration scenario, and ≥30% percentage of native vegetation restoration scenario (d). This figure was elaborated by the first author using softwares ArcGIS 10.1 and IrfanView 4.37.

The ensemble predictions showed a continuous potential distribution for pumas in São Paulo State, Brazil. All model outputs had a high discriminative ability with an average AUC of 0.84 ± 0.02 for the testing data and 0.88 ± 0.02 for the training data. Maxent’s average model logistic output revealed a continuous distribution of pumas in São Paulo State (Fig 2). Table 2 shows the model results of low, medium and high habitat suitability and the cross tabulated area analysis with different land cover types (see S5 Table for protection levels cross tabulation analysis).

**Table 2:** Cross-tabulated areas between the four classes of *Puma concolor* habitat suitability (HS) (i.e. low HS (values ≤ 0.17), medium HS (0.17 ≤ values ≤ 0.31), medium-high HS (0.31 ≤ values ≤ 0.50) and high HS (values > 0.50) and four land cover zones (i.e. native vegetation, exotic forest crops, agriculture and others—urban areas and water bodies pixels) calculated using ArcGIS 10.1 Spatial Analyst Zonal tool.

Land cover	Low HS (km^2^)	Medium HS (km^2^)	Medium-high HS (km^2^)	High HS (km^2^)
**Native vegetation**	16719 (26%)	11589 (10%)	9888 (17%)	9288 (70%)
**Forest crops**	1468 (2%)	2529 (2%)	2802 (5%)	974 (7%)
**Agriculture**	38612 (60%)	94284 (83%)	42656 (75%)	2861 (21%)
**Other**	8093 (12%)	4379 (5%)	1392 (3%)	113 (2%)
**Total in São Paulo**	64892 (100%)	112781 (100%)	56739 (100%)	13237 (100%)

**Fig 3:**
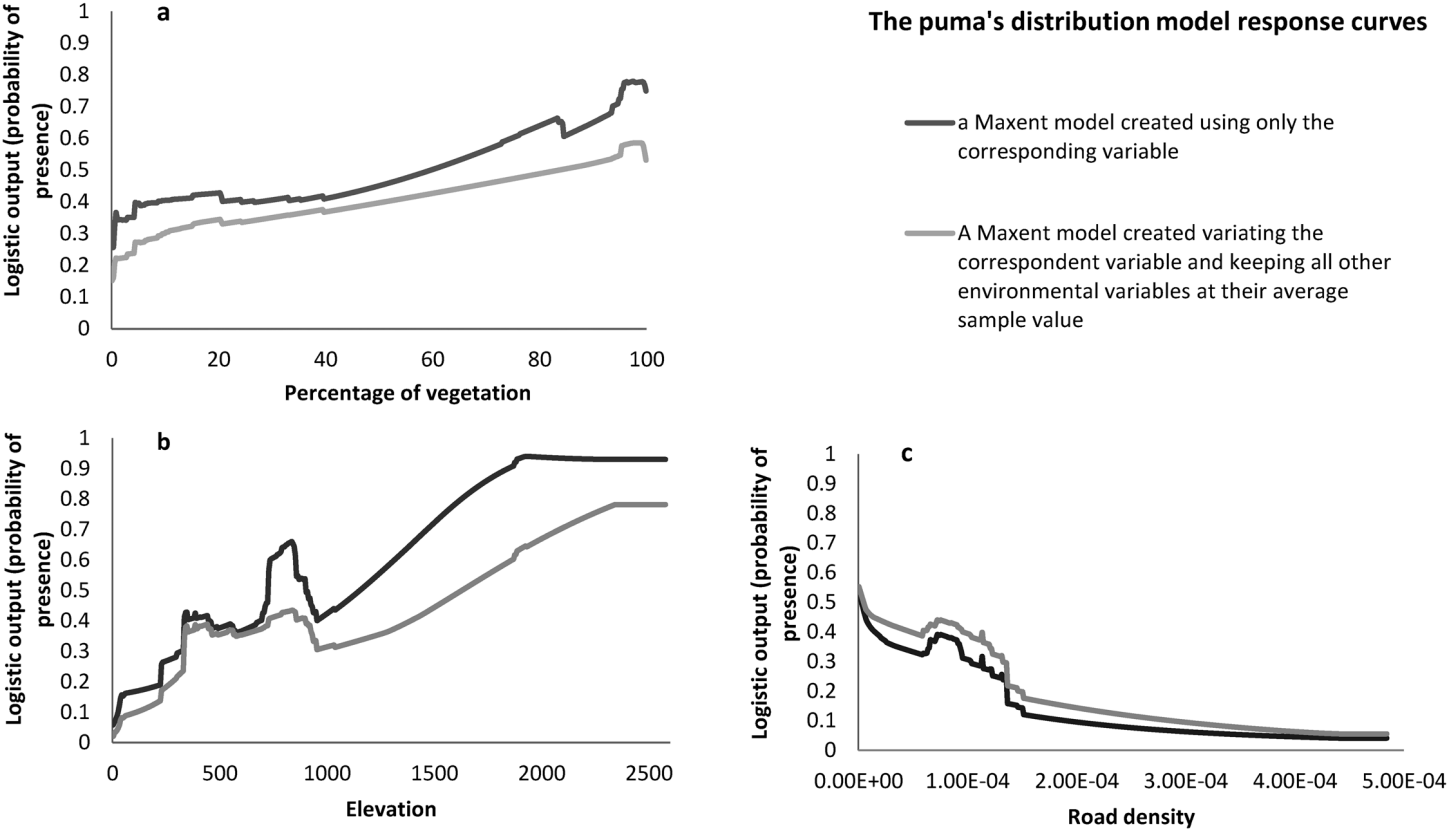
Marginal response curves showing how the logistic prediction changed as each of the three environmental variables that contributed the most to the models were varied: native vegetation (a), elevation (b) and density of roads (c).

The puma’s distribution was positively influenced by percentage of native vegetation (Fig 3a), elevation (Fig 3b) percentage of exotic forest crops and slope. It responded negatively to road density (Fig 3c), watercourse density and edge density of native vegetation. Percentage of native vegetation, elevation and density of roads were considered the three most important environmental variables to the model prediction. They had the highest relative contributions, the highest regularized training gain when used in isolation in the Jackknife test and also the highest AUC values when used in isolation. Percentage of native vegetation had the highest permutation importance and the highest test gain when used in isolation in the Jackknife test. Elevation dropped regularized training gain the most when omitted in the modeling process. Road density dropped the test gain the most when omitted in the modeling process (Table 3).

**Table 3:** Environmental variable importance to the modeling process evaluated by percent contribution, permutation importance and training gain (Jackknife test). Three highest values are indicated in bold.

Variable	Percent contribution	Permutation importance	Training gain with only the variable	Drop in training gain without the variable	Test gain with only the variable	Drop in test gain without the variable
**Percentage of native vegetation**	**23.61**	**37.09**	**0.24**	0.05	**0.54**	**0.08**
**Elevation**	**26.49**	11.38	**0.28**	**0.15**	**0.34**	**0.06**
**Road density**	**23.98**	**16.19**	**0.25**	**0.08**	0.29	**0.09**
**Slope**	7.11	8.50	0.03	**0.07**	0.05	0.05
**Watercourse density**	5.01	4.98	0.13	0.04	0.23	0.04
**Percentage of exotic forest crops**	7.45	**12.59**	0.11	**0.07**	0.16	**0.08**
**Edge density**	6.34	9.27	0.23	0.02	**0.47**	0.03

**Fig 4:**
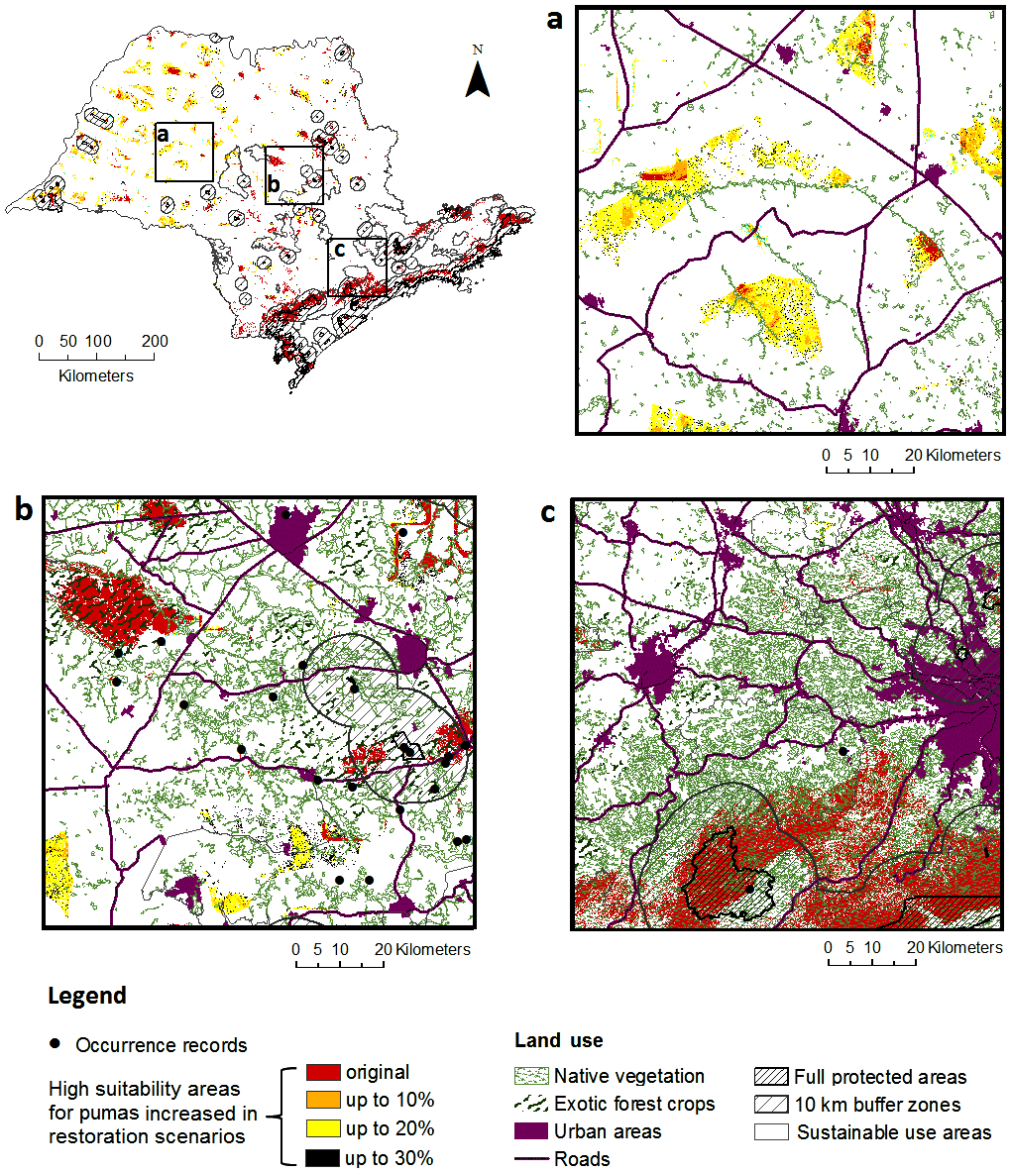
High probability of puma presence (original) in São Paulo State, Brazil and high probability of puma presence projected in three restoration scenarios (≥10% percentage of native vegetation, ≥20% percentage of native vegetation, and ≥30% percentage of native vegetation) zoomed in for a close-up of three different landscape regions: (a) Northwestern region, (b) Central region and (c) Southeastern region. This figure was elaborated by the first author using softwares ArcGIS 10.1 and IrfanView 4.37.

Maxent’s projections of predicted puma’s habitat suitability in restoration scenarios showed an increase of high suitability areas for pumas (>0.5) in all novel environments (percentage of native vegetation value ≤ 10%, ≤ 20%, ≤ 30%) compared to the original species distribution scenario (Fig 4). The new high suitability areas occur especially in Northeastern (Fig 4a), and also in Central region (Fig 4b) of São Paulo State. The results showed no new high suitability areas emerging in the Coastal region and São Paulo City Metropolitan region (Fig 4c).

The SDM projection to scenario 1 (≥10% native vegetation cover) showed 15637 km^2^ as high suitability areas for pumas (probability of presence values > 0.50), an increase of 2400km^2^ (18%) in the original high suitability areas for pumas (13236 km^2^). These new high suitability areas have emerged especially in highly deforested areas in Northeastern region of São Paulo State (Fig 4a).

The SDM projection to scenario 2 (≥20% native vegetation cover) showed 21 404 km^2^ as high suitability areas for pumas (probability of presence values > 0.50), an increase of 8167 km^2^ (62%) in the high suitability areas for pumas. It has a 5767 km^2^ additional difference of high suitability areas comparing to the scenario 1 (≥10% native vegetation cover). These new high suitability areas have emerged particularly in Northeastern (Fig 4a) and Central region (Fig 4b).

The SDM projection to scenario 3 (≥30% native vegetation cover) showed 22 800 km^2^ as high suitability areas for pumas, an increase of 9564 km^2^ (72%) in the high suitability areas for pumas. It has a 1396 km^2^ additional difference of high suitability areas comparing to the scenario 2 (≥20% native vegetation cover). The new high suitability areas have not emerged clustered in new regions. The additional pixels with probability of presence values > 0.50 have been spread in the landscape around areas emerged in scenarios 1 and 2.

## Discussion

### Habitat suitability and environmental variables

Although São Paulo has a high level of habitat loss and fragmentation, the models generated in this study showed the puma still has a continuous potential distribution in the state. However, there were significant differences in São Paulo State territory with respect to the puma’s habitat suitability. These differences were particularly due to differing levels of fragmentation and habitat loss (represented by percentage of native vegetation response), human induced land use changed (represented by road density response) and topography (represented by the elevation response) in São Paulo State.

Despite the puma’s high capacity to adapt to various environments and not a strictly forest dependent species (Occurrence records found from 0 to 100% native vegetation–S1 Table), the high positive relationship between pumas and native vegetation showed in this study concurs with a previous study that found that the puma’s habitat contains proportionally more natural habitat than modified land cover types [24]. This confirms that habitat loss as the major driver of population decline of the puma (sensu [62]). Nevertheless, rural areas in São Paulo State are highly suitable for this adaptable cat in this study, especially nearby native vegetation patches (e.g. São Paulo metropolitan region–Fig 4c). Indeed, there is evidence in Southern California that pumas incorporate rural and peri-urban development areas into their home ranges, apparently perceiving these areas as non-habitat [57]. In the study area, there is evidence that pumas consume complementary resources derived from the agricultural matrix in Campinas Metropolitan Region and Ribeirão Preto, São Carlos and Rio Claro Region [80].

The strong positive relationship between puma probability of presence and elevation might be responsible for the lower values in the South and Coast region. Although the native vegetation cover is higher than 30% in the São Paulo South and Coast region, the modelling showed very low suitability for pumas (probability of presence ≤ 0.1) in these areas (S2 Fig). Although low elevation does not limit species presence (Occurrence records found from 0 to 1934 m elevation–S1 Table), the data used has shown few records in low elevations (five presence records from 0 to 100 m and six presence records from 100 to 300 m). This may be because pumas have used higher elevations more often than lower elevations in the study area, as was reported in South California [57]. In addition, there are urban areas and major roads in these regions that could be influencing the final habitat suitability map (represented by the road density variable). Further investigation is necessary to confirm and clarify the low suitability for pumas in this area.

The strong negative relationship between puma probability of presence and road density (Occurrence records found from 0 to 1.56 x 10^−4^ m/m^2^ –S1 Table) agrees with previous studies indicating that pumas tend to avoid paved roads [81] and also indirectly confirms evidence that pumas tend to avoid human-disturbed areas [28]. In this study, higher road densities are correlated to urban areas, representing not only dispersion but also human settlements and habitat availability (S2 Table). Although our modelling highlights the low probability of puma presence in the São Paulo and Campinas metropolitan region, the most developed in the study area, high suitability areas were found in their surroundings when covered by native vegetation patches (S3 Fig).

It is necessary to be aware that the puma’s responses to the environmental variables could change in different regions and landscape configurations (e.g., regions with more native vegetation habitats than human-modified land as occurs in the Amazon). Combining datasets over large spatial scales is necessary to generate global models for the species and to infer the relative importance of different areas for puma conservation. Long-term species monitoring programs are also crucial to improve the accuracy of the distribution modelling process.

### Restoration scenarios

Currently, there is noclearly defined threshold limiting regardinghow much native vegetation is suitable for the maintenance of puma populations. We therefore based our restoration scenarios on ecological assumptions (e.g. [59], [60], [61], [62]) and the environmental legislation [63]. Nevertheless, nothing guarantees that restored areas will be occupied by pumas.

The projected impacts of habitat restoration for pumas were positive, improving the habitat suitability for the species in Central and North-western region and maintaining the suitability in Coastal and South regions (Fig 4). The restoration benefit varies both in shape and rate of change considering each restoration scenario. Restoration ≥20% native vegetation has the highest restoration improvement rate in all evaluated scenarios, doubling the improvement rate of the ≥10% scenario. Surprisingly, ≥30% restoration scenario showed almost no improvement in the on the puma’s habitat suitability compared to ≥20% restoration scenario. This reflects the large reduction in the distance among patches between the 10–20% and 20–30% restoration scenarios [61].

This study used simplified restoration scenarios to explore the habitat suitability response when habitat restoration is projecting in high fragmented human-modified landscapes. It is important to realize that the alternative scenarios do not pretend to establish future population dynamics for the puma in the study region. While Maxent modeling for the current land use configuration evaluated the original model performance very well, it was assumed that the restoration scenarios projections based on the original model were also well predicted. Araújo et al. [82] questioned this assumption (especially for projections under climate change scenarios) arguing the evaluation procedure does not incorporate uncertainty in the projections. Furthermore, the land use based models implemented in this study make a number of simplifying assumptions that may bias the projections [83]. Exploring these represent important avenues for future researches (e.g. [84]).

No method was applied for determining which future is more likely. For example, this study do not face important challenges of the restoration process, such as detecting barriers regarding locations to restore [85], evaluating cost-effective outcomes [86], integrating biological and social-economic values [87] and conforming to socio-political issues. In addition, a conservation planning approach incorporating multiple actions (e.g. restoration, reversing defaunation, conflict management, protected areas implementation, certified timber management) would provide insights about best solutions for the species conservation. These are important focuses for further research.

### Conservation planning implications

The results presented in this study reinforce the argument that although large protected areas are usually the main strategy to safeguard biodiversity, native vegetation on private lands has an important conservation value [88]. These small patches extend the amount of habitat available for puma, increase landscape connectivity and possibly facilitate dispersion movements by acting as stepping-stone habitats for pumas (e.g., [43]). Importantly, these results highlight the importance of private proprieties as an essential habitat component for pumas, such as shown for amphibians [89] and birds [64] in Brazil.

Only 24% (~3189 km^2^) of the total high suitability areas for pumas are fully protected by law in São Paulo State (S3 Table). Therefore, high suitability areas for pumas might be considered for new full protected areas sites and also for expanding the existing protected areas. However, the diffuse high suitability areas for pumas shown in this study suggests that a new conservation approach is necessary in tropical rural landscapes, beyond the protection of few areas minimally impacted by past or present human activities [2]. In addition, areas in a 10 km buffer distance from fully protected areas are especially important, comprising an additional 33% (~4 389 km^2^) of high suitability areas for pumas (S3 Table). This transition zone should be considered in the protected areas management plans [90] as the minimum necessary to accommodate edge effects for the species [43].

The restoration scenarios indicate the need to effectively adhere the Brazilian Forest Code [63], restoring at least 20% of private land in São Paulo State in the agri-environment scheme called Legal Reserve for improving habitat suitability for pumas. In 2010, approximately 98% of the private rural proprieties in São Paulo State did not comply with this mandatory practice [91]. Locating offset sites for Legal Reserves out of the State should be avoided even if located in the same biome [92], considering the restoration needs. Furthermore, agriculture activities should be managed and regulated in order to reduce their negative impact on pumas (e.g. Agro-environmental zoning for the Sugar and Alcohol Sector). In addition, economic instruments such as payment for environmental services (PES) must be encouraged in order to enhance the restoration process (e.g. the Puma Corridor proposed to conserve the water and biodiversity in Campinas region). Surely, the maintenance and improvement of conservation policies in national and regional scales are crucial for restoration scenarios to become reality.

Usually, species responses to land use change scenarios that destroy habitat in urban and agricultural landscapes should be assessed by EIA and SEA processes [93]. In a similar procedure, an approach applied here that estimates species responses to habitat restoration process, could be incorporated into environmental law procedures. Studies such as that presented here need to inform revisions in the legislation and minimize risks of environmental setbacks with critical and irreversible consequences. One such example occurred recently in the Brazilian Forest Code [63] when the legislation was considered overprotective of the environment by political reviewers [94].

## Conclusions

This case study highlights the importance of a landscape planning approach that includes not only the establishment and management of protected areas, but also restoration and protection of habitat on private lands for the conservation of pumas. In addition, it shows how specific native vegetation restoration targets might improve the suitability of areas for pumas and highlights existing legal mechanisms to promote landscape recovery in São Paulo State, Brazil. Importantly, the results directly inform the decision-making process by guiding environmental policies and land use planning, and assisting species’ conservation. They serve as a useful model to guide similar process for other large-carnivore species world-wide.

## Supporting Information

S1 Fig Gaussian weighted bias grid surface for puma’s occurrence records in São Paulo State. This figure was elaborated by the first author using software ArcGIS 10.1.
(TIF)

S2 Fig Very low habitat suitability (≤ 0.1 probability of presence) corresponding to low elevation rate (≤ 100 meters) in São Paulo State South and Cost region. This figure was elaborated by the first author using softwares ArcGIS 10.1 and IrfanView 4.37.
(TIF)

S3 Fig Very low habitat suitability (≤ 0.1 probability of presence) and high habitat suitability (≥ 0.5 probability of presence) in São Paulo City Metropolitan region. This figure was elaborated by the first author using softwares ArcGIS 10.1 and IrfanView 4.37.
(TIF)

S1 Table Localities and environmental variables values for the 342 puma’s occurrence records. The values are from each environmental variable pixel coincident with the occurrence records points of the puma (*Puma concolor*) in São Paulo State used for SDM. Latitude (LAT) and Longitude (LONG) in decimal degrees, South America Albers Equal Area Conic Projection). Species Distribution Model (SDM), Percent of native vegetation (VEG), edge density (EDG), forest crops (FOR), road density (ROAD), Elevation (ELV), Slope (SLO) and Water density (WAT).
(DOCX)

S2 Table Ecological relationships relevant for pumas published in the literature that were considered as potential explanatory variables (n = 13) to guide the species distribution modelling (SDM). (DOCX)

S3 Table Pearson’s correlation analysis of the environmental variables. (DOCX)

S4 Table Environmental variables not used to develop the models for pumas in São Paulo State, Brazil. All variables were considered correlated to others (Pearson’s correlation ≥±0.5).
(DOCX)

S5 Table Cross-tabulated areas calculated using ArcGIS 10.1 Spatial Analyst Zonal tool between the four probability of *Puma concolor* presence classes (i.e. low (values ≤ 0.17), medium (0.17 ≤ values ≤ 0.31), medium-high (0.31 ≤ values ≤ 0.50 and high (values > 0.50) habitat suitability and full protected areas (FPA), 10 km buffer zone from full protected areas (10KM), sustainable reserves (Áreas de Proteção Ambiental—APA) and Non-Protected Areas (NPA). (DOCX)
