# Supplementary figures and images for: Using Species Distribution Models to Predict Potential Landscape Restoration Effects on Puma Conservation
Source: PLoS One. 2016 Jan 6;11(1):e0145232. doi: 10.1371/journal.pone.0145232 (PMC4703218; doi:10.1371/journal.pone.0145232)

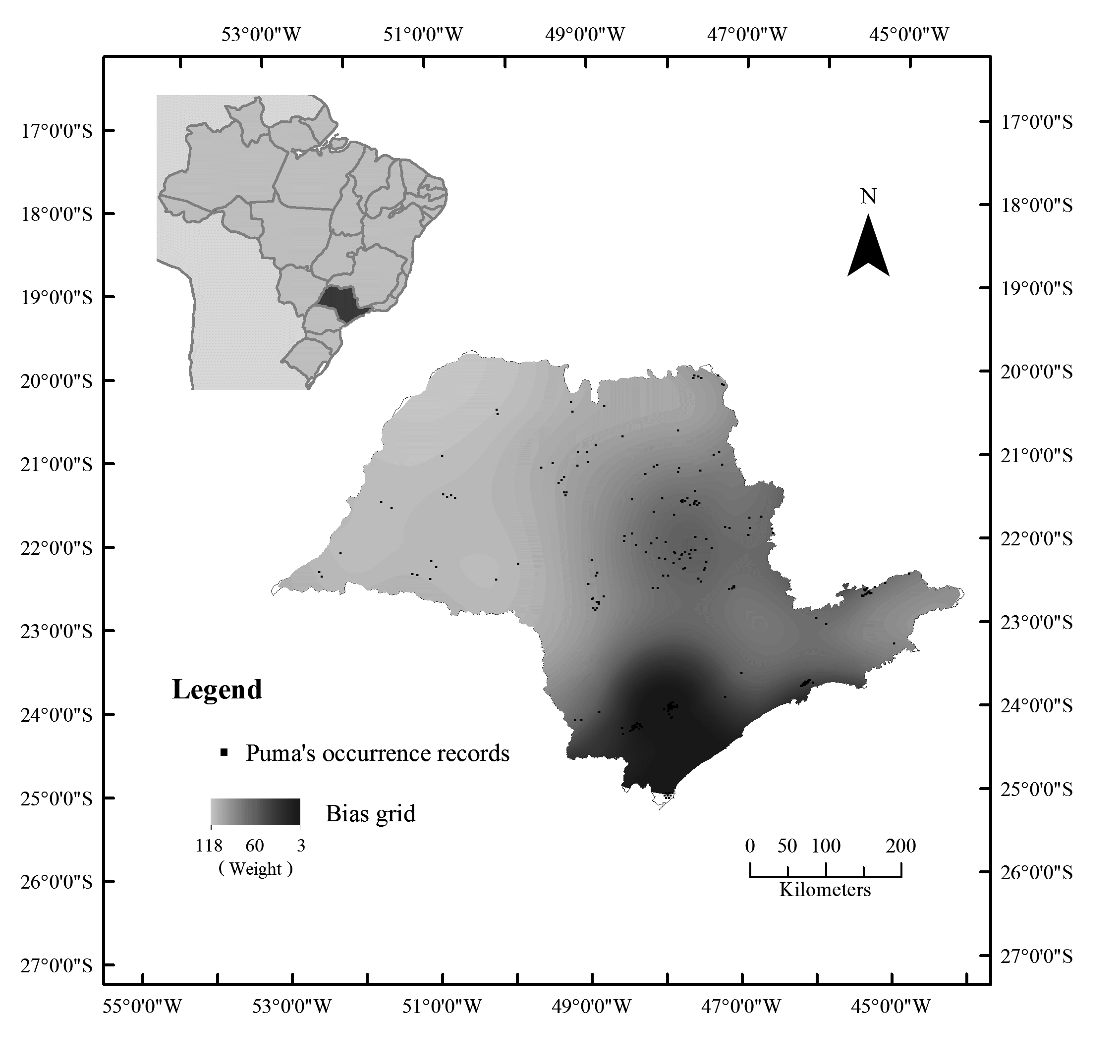

Supplement: S1 Fig — This figure was elaborated by the first author using software ArcGIS 10.1. (TIF) [file pone.0145232.s001.tif]

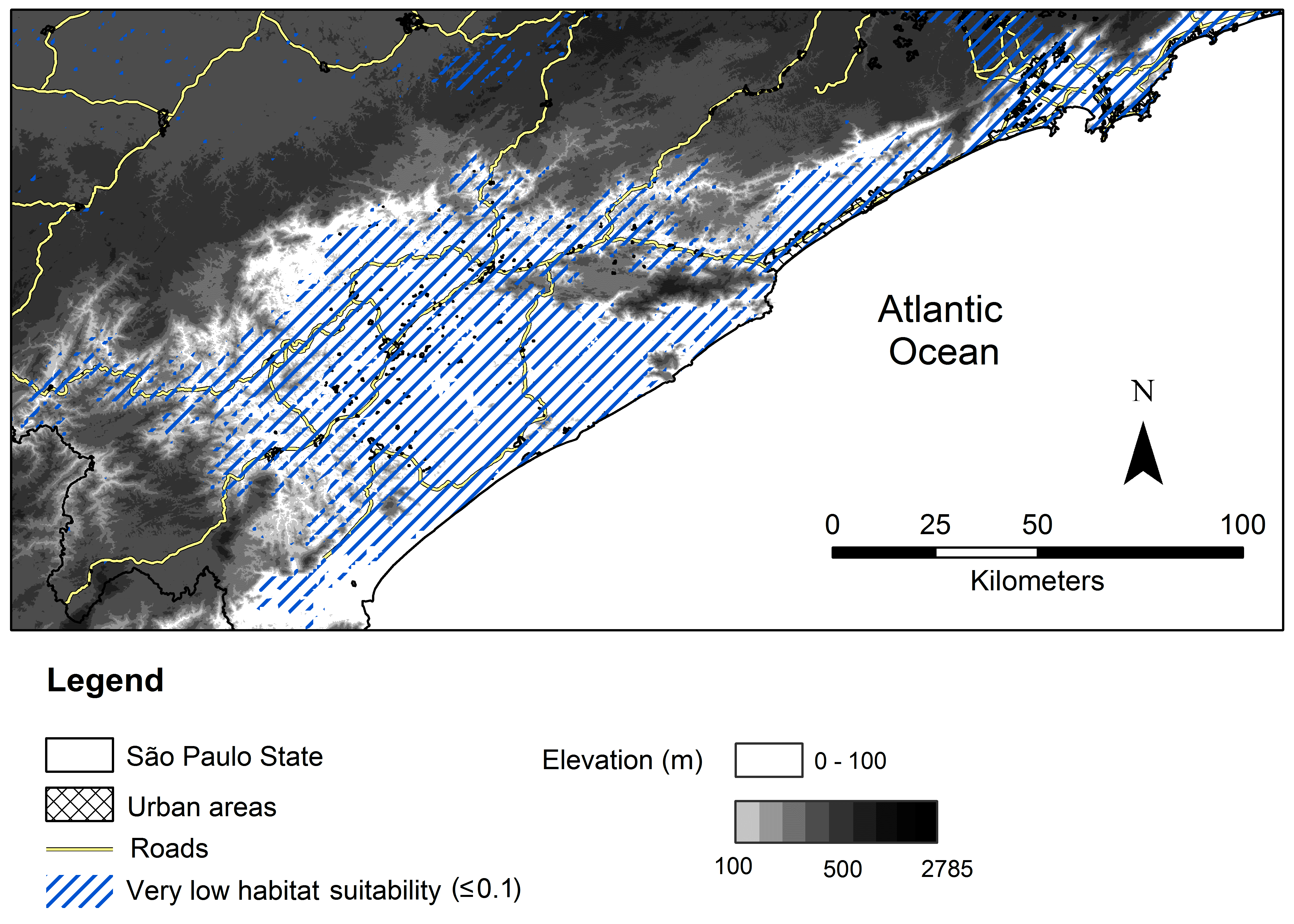

Supplement: S2 Fig — This figure was elaborated by the first author using softwares ArcGIS 10.1 and IrfanView 4.37. (TIF) [file pone.0145232.s002.tif]

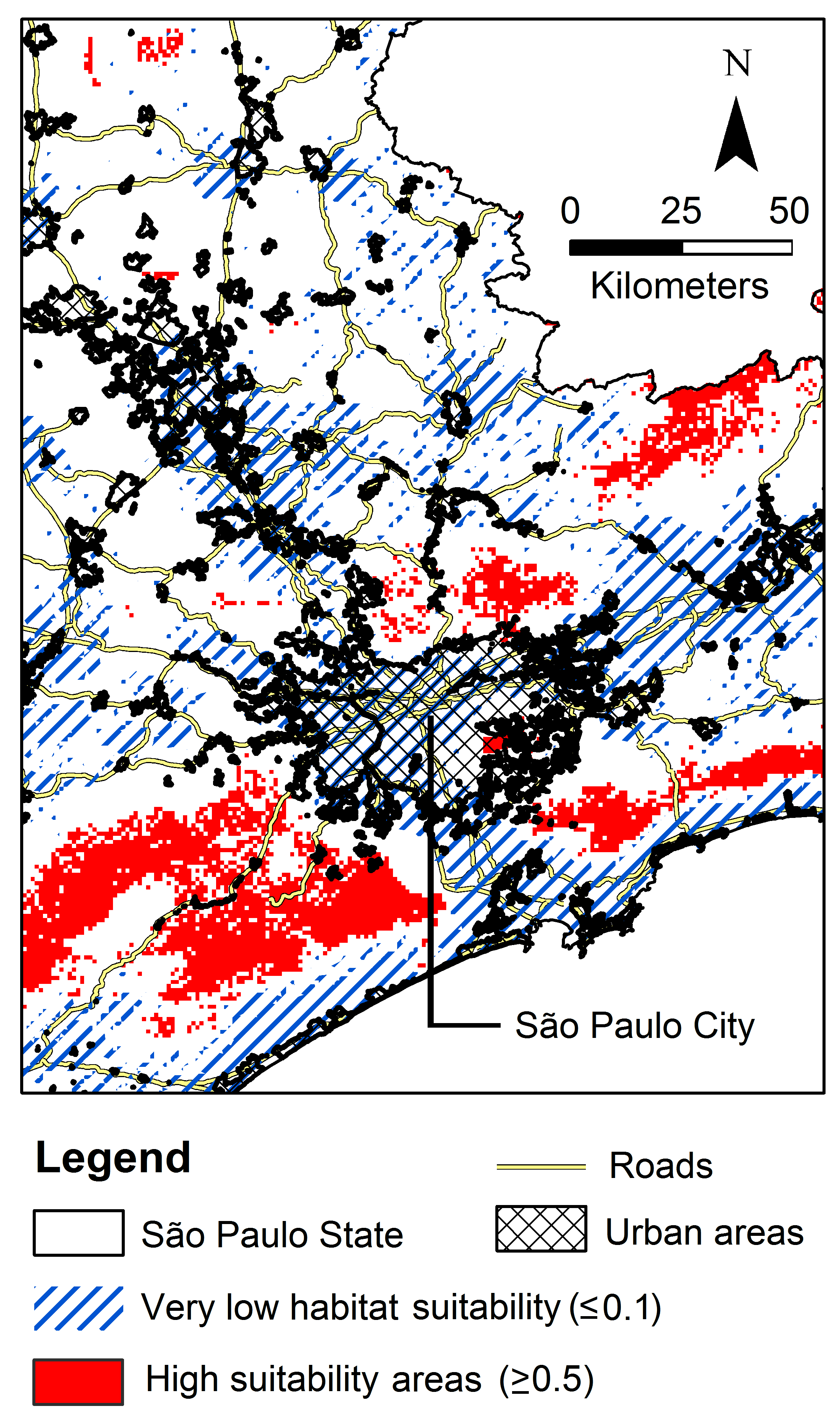

Supplement: S3 Fig — This figure was elaborated by the first author using softwares ArcGIS 10.1 and IrfanView 4.37. (TIF) [file pone.0145232.s003.tif]
