# Supplementary material for: Using Species Distribution Models to Predict Potential Landscape Restoration Effects on Puma Conservation
Source: PLoS One. 2016 Jan 6;11(1):e0145232. doi: 10.1371/journal.pone.0145232 (PMC4703218; doi:10.1371/journal.pone.0145232)
Supplement: S1 Table — The values are from each environmental variable pixel coincident with the occurrence records points of the puma (Puma concolor) in São Paulo State used for SDM. Latitude (LAT) and Longitude (LONG) in decimal degrees, South America Albers Equal Area Conic Projection). Species Distribution Model (SDM), Percent of native vegetation (VEG), edge density (EDG), forest crops (FOR), road density (ROAD), Elevation (ELV), Slope (SLO) and Water density (WAT). (DOCX) [file pone.0145232.s004.docx]

Table S1. Localities and environmental variables values for the 342 puma’s occurrence records. The values are from each environmental variable pixel coincident with the occurrence records points of the puma (*Puma concolor*) in São Paulo State used for SDM. Latitude (LAT) and Longitude (LONG) in decimal degrees, South America Albers Equal Area Conic Projection). Species Distribution Model (SDM), Percent of native vegetation (VEG), edge density (EDG), forest crops (FOR), road density (ROAD), Elevation (ELV), Slope (SLO) and Water density (WAT).

| **ID** | **LAT** | **LONG** | **SDM** | **VEG** | **EDG** | **FOR** | **ROAD** | **ELEV** | **SLO** | **WAT** |
| --- | --- | --- | --- | --- | --- | --- | --- | --- | --- | --- |
| 1 | -22.708717 | -45.437153 | 0.949929 | 81.8472 | 0.002002 | 0 | 0 | 1934 | 4.99385 | 0.00025 |
| 2 | -22.617029 | -45.316305 | 0.949418 | 90.1597 | 0.002002 | 0 | 0 | 1883 | 16.5724 | 0.000147 |
| 3 | -22.707603 | -45.432747 | 0.937042 | 81.5625 | 0.002002 | 0 | 0 | 1918 | 11.5315 | 0.00025 |
| 4 | -22.688776 | -45.368719 | 0.935674 | 83.8958 | 0.002002 | 0 | 0 | 1913 | 1.9133 | 0.000245 |
| 5 | -22.6975 | -45.42031 | 0.927131 | 79.9653 | 0.002002 | 0 | 0 | 1927 | 12.0097 | 0.000238 |
| 6 | -22.728574 | -45.476589 | 0.920779 | 84.0833 | 0.002002 | 0 | 0 | 1874 | 18.7525 | 0.000264 |
| 7 | -22.719414 | -45.475829 | 0.919058 | 83.0347 | 0.002002 | 0 | 0 | 1926 | 22.5107 | 0.000268 |
| 8 | -22.691831 | -45.378341 | 0.918026 | 82.6319 | 0.002002 | 0 | 0 | 1875 | 16.3388 | 0.000251 |
| 9 | -22.718952 | -45.47951 | 0.911778 | 82.4722 | 0.002002 | 0 | 0 | 1917 | 23.5596 | 0.000269 |
| 10 | -24.0986 | -47.975535 | 0.911257 | 96.3264 | 0.001007 | 0.935728 | 0 | 739 | 9.92336 | 0.000326 |
| 11 | -22.725163 | -45.462468 | 0.909318 | 83.5486 | 0.002002 | 0 | 0 | 1913 | 24.0165 | 0.000263 |
| 12 | -22.688194 | -45.361532 | 0.9079 | 83.4514 | 0.002002 | 0 | 0 | 1845 | 16.6041 | 0.000248 |
| 13 | -22.721668 | -45.47557 | 0.905934 | 83.1736 | 0.002002 | 0 | 0 | 1928 | 26.6646 | 0.000267 |
| 14 | -24.059961 | -47.974679 | 0.898559 | 77.0208 | 0.001007 | 0.908408 | 0 | 819 | 4.8071 | 0.000347 |
| 15 | -24.31233 | -48.452014 | 0.891094 | 97.0347 | 0.001007 | 0.054641 | 0 | 734 | 11.4518 | 0.000336 |
| 16 | -22.72385 | -45.474411 | 0.89032 | 83.4375 | 0.002002 | 0 | 0 | 1916 | 32.2669 | 0.000265 |
| 17 | -24.284873 | -48.376624 | 0.888108 | 98.7361 | 0.001007 | 0.034151 | 0 | 817 | 15.7833 | 0.000328 |
| 18 | -24.280882 | -48.385248 | 0.887722 | 98.0694 | 0.001007 | 0.034151 | 0 | 789 | 15.1939 | 0.000336 |
| 19 | -24.063028 | -47.975299 | 0.884718 | 79.0764 | 0.001007 | 0.894748 | 0 | 836 | 8.93328 | 0.000354 |
| 20 | -24.057347 | -47.969439 | 0.88461 | 77.5278 | 0.001007 | 0.259545 | 0 | 826 | 3.87413 | 0.000345 |
| 21 | -22.647231 | -45.455124 | 0.881058 | 82.75 | 0.002002 | 0 | 0 | 1556 | 11.2584 | 0.000186 |
| 22 | -24.272958 | -48.391487 | 0.879347 | 96.5208 | 0.001007 | 0.034151 | 0 | 814 | 18.5748 | 0.00033 |
| 23 | -24.060713 | -47.974607 | 0.879137 | 77.6806 | 0.001007 | 0.846937 | 0 | 816 | 9.2553 | 0.000351 |
| 24 | -22.671451 | -45.388863 | 0.878204 | 82.9028 | 0 | 0 | 0 | 1895 | 5.88553 | 0.000211 |
| 25 | -24.084024 | -47.949471 | 0.876102 | 98.0417 | 0.001007 | 0 | 0 | 835 | 7.17487 | 0.00033 |
| 26 | -23.775107 | -46.175377 | 0.870356 | 96.2639 | 0.001007 | 0.949389 | 0.000073 | 748 | 3.23709 | 0.000317 |
| 27 | -22.685531 | -45.403556 | 0.869731 | 81.0694 | 0 | 0 | 0 | 1914 | 2.61114 | 0.000227 |
| 28 | -24.085227 | -47.944777 | 0.867732 | 98.8333 | 0.001007 | 0 | 0 | 789 | 9.12083 | 0.00033 |
| 29 | -24.085794 | -47.964866 | 0.867212 | 96.3958 | 0.001007 | 0 | 0 | 743 | 4.2996 | 0.000344 |
| 30 | -23.775858 | -46.175294 | 0.86705 | 96.1667 | 0.001007 | 0.949389 | 0.000073 | 741 | 5.75582 | 0.000317 |
| 31 | -24.067337 | -47.937349 | 0.86628 | 95.5972 | 0.001007 | 0 | 0 | 835 | 9.66405 | 0.000339 |
| 32 | -24.071638 | -47.922285 | 0.865869 | 99.5972 | 0.001007 | 0 | 0 | 847 | 8.1876 | 0.000331 |
| 33 | -24.055064 | -47.957758 | 0.864814 | 81.5208 | 0.001007 | 0.00683 | 0 | 848 | 10.5665 | 0.000343 |
| 34 | -24.071367 | -47.929636 | 0.861619 | 98.8889 | 0.001007 | 0 | 0 | 767 | 2.39162 | 0.000338 |
| 35 | -24.042617 | -47.952552 | 0.858994 | 75.9028 | 0.001007 | 0.00683 | 0 | 827 | 8.68656 | 0.000329 |
| 36 | -24.301759 | -48.344788 | 0.858987 | 99.9583 | 0.001007 | 0 | 0 | 841 | 4.21903 | 0.000341 |
| 37 | -23.773261 | -46.17101 | 0.857508 | 96.3403 | 0.001007 | 0.949389 | 0.000078 | 752 | 5.15171 | 0.000313 |
| 38 | -24.080924 | -47.959842 | 0.857191 | 95.7986 | 0.001007 | 0 | 0 | 741 | 2.87791 | 0.000338 |
| 39 | -24.059841 | -47.97286 | 0.856703 | 78.2222 | 0.001007 | 0.594222 | 0 | 811 | 13.7188 | 0.000346 |
| 40 | -24.040361 | -47.952769 | 0.856383 | 74.0208 | 0.001007 | 0.00683 | 0 | 817 | 5.3862 | 0.000324 |
| 41 | -24.073502 | -47.927598 | 0.855574 | 99.5764 | 0.001007 | 0 | 0 | 807 | 10.3764 | 0.000345 |
| 42 | -24.085647 | -47.951146 | 0.854012 | 98.2292 | 0.001007 | 0 | 0 | 801 | 2.02936 | 0.00033 |
| 43 | -24.094188 | -47.942996 | 0.853853 | 99.875 | 0.001007 | 0 | 0 | 833 | 3.73583 | 0.000324 |
| 44 | -23.773674 | -46.17645 | 0.853304 | 96.5069 | 0.001007 | 0.949389 | 0.000072 | 758 | 6.4352 | 0.000311 |
| 45 | -24.08757 | -47.95737 | 0.850564 | 98.2986 | 0.001007 | 0 | 0 | 790 | 12.2585 | 0.000334 |
| 46 | -23.80353 | -46.210643 | 0.850367 | 97.4653 | 0.001007 | 0.949389 | 0.00008 | 768 | 5.10262 | 0.000277 |
| 47 | -24.069228 | -47.954561 | 0.85034 | 92.2222 | 0.001007 | 0 | 0 | 855 | 3.50844 | 0.000329 |
| 48 | -24.037294 | -47.95215 | 0.849869 | 70.3333 | 0.001007 | 0.00683 | 0 | 816 | 7.01687 | 0.00033 |
| 49 | -24.104162 | -47.979578 | 0.848357 | 96.0208 | 0.001007 | 1.98074 | 0 | 743 | 8.87418 | 0.000321 |
| 50 | -22.629331 | -45.411865 | 0.847507 | 81.3889 | 0 | 0 | 0 | 1776 | 9.97854 | 0.000152 |
| 51 | -24.089466 | -47.951693 | 0.846865 | 99.0486 | 0.001007 | 0 | 0 | 801 | 1.21008 | 0.000331 |
| 52 | -24.034287 | -47.95244 | 0.842758 | 67.4236 | 0.001007 | 0.047811 | 0 | 779 | 6.17391 | 0.000331 |
| 53 | -23.785506 | -46.222688 | 0.841444 | 97.1181 | 0.001007 | 0.949389 | 0.000015 | 909 | 4.59291 | 0.000252 |
| 54 | -24.069887 | -47.964568 | 0.840513 | 89.5347 | 0.001007 | 0 | 0 | 844 | 5.68382 | 0.000328 |
| 55 | -24.06499 | -47.947646 | 0.837009 | 91.9583 | 0.001007 | 0 | 0 | 754 | 5.75979 | 0.000347 |
| 56 | -24.080711 | -47.968103 | 0.836382 | 93.7361 | 0.001007 | 0 | 0 | 754 | 7.3606 | 0.000343 |
| 57 | -24.039609 | -47.952842 | 0.836281 | 72.3056 | 0.001007 | 0.00683 | 0 | 808 | 9.76532 | 0.000327 |
| 58 | -24.072991 | -47.93131 | 0.836103 | 98.8542 | 0.001007 | 0 | 0 | 812 | 16.8666 | 0.000339 |
| 59 | -23.785765 | -46.186086 | 0.835955 | 96.0764 | 0.001007 | 0.949389 | 0.000063 | 748 | 2.73942 | 0.000309 |
| 60 | -24.064841 | -47.933929 | 0.835758 | 95.6458 | 0.001007 | 0 | 0 | 793 | 14.1328 | 0.000337 |
| 61 | -24.075998 | -47.931019 | 0.835592 | 99.375 | 0.001007 | 0 | 0 | 807 | 15.1032 | 0.000348 |
| 62 | -24.051878 | -47.95532 | 0.835301 | 80.1806 | 0.001007 | 0.00683 | 0 | 840 | 16.4059 | 0.000341 |
| 63 | -24.06884 | -47.937204 | 0.834681 | 96.4236 | 0.001007 | 0 | 0 | 818 | 16.5143 | 0.000335 |
| 64 | -23.798958 | -46.220291 | 0.834647 | 97.4861 | 0.001007 | 0.949389 | 0.000049 | 799 | 4.45124 | 0.000261 |
| 65 | -24.094368 | -47.945725 | 0.834085 | 99.875 | 0.001007 | 0 | 0 | 862 | 5.65152 | 0.000323 |
| 66 | -24.081828 | -47.950599 | 0.833232 | 97.3264 | 0.001007 | 0 | 0 | 842 | 17.9292 | 0.000331 |
| 67 | -24.061863 | -47.946117 | 0.833102 | 90.0278 | 0.001007 | 0 | 0 | 748 | 4.57794 | 0.000346 |
| 68 | -23.797178 | -46.206771 | 0.832383 | 96.5833 | 0.001007 | 0.949389 | 0.000067 | 758 | 2.42015 | 0.000277 |
| 69 | -24.086938 | -47.959262 | 0.832098 | 97.6667 | 0.001007 | 0 | 0 | 809 | 17.5735 | 0.000337 |
| 70 | -24.076898 | -47.944666 | 0.832081 | 97.25 | 0.001007 | 0 | 0 | 768 | 14.1458 | 0.000335 |
| 71 | -24.054519 | -47.972457 | 0.831643 | 73.1806 | 0.001007 | 0.717164 | 0 | 789 | 17.9891 | 0.000343 |
| 72 | -24.079393 | -47.948087 | 0.831603 | 97.1875 | 0.001007 | 0 | 0 | 797 | 16.937 | 0.000335 |
| 73 | -24.087303 | -47.94183 | 0.826944 | 99.2083 | 0.001007 | 0 | 0 | 780 | 16.2701 | 0.000331 |
| 74 | -24.060691 | -47.939822 | 0.82693 | 91.6597 | 0.001007 | 0 | 0 | 832 | 11.7263 | 0.000342 |
| 75 | -24.082275 | -47.968868 | 0.825764 | 94.1528 | 0.001007 | 0 | 0 | 780 | 11.4798 | 0.000346 |
| 76 | -22.660781 | -45.444497 | 0.824339 | 83.3819 | 0.002002 | 0 | 0 | 1552 | 20.0086 | 0.000211 |
| 77 | -24.044872 | -47.952334 | 0.823553 | 77.4653 | 0.001007 | 0.00683 | 0 | 820 | 16.8829 | 0.000334 |
| 78 | -23.766295 | -46.169038 | 0.823234 | 96.0139 | 0.001007 | 0.949389 | 0.000077 | 754 | 10.5166 | 0.0003 |
| 79 | -23.771547 | -46.1584 | 0.819915 | 95.3333 | 0.001007 | 0.949389 | 0.00009 | 850 | 10.4095 | 0.000292 |
| 80 | -23.775996 | -46.177108 | 0.818875 | 96.3889 | 0.001007 | 0.949389 | 0.000071 | 745 | 12.8366 | 0.000317 |
| 81 | -23.768204 | -46.164255 | 0.818221 | 95.8194 | 0.001007 | 0.949389 | 0.000086 | 732 | 8.6125 | 0.000293 |
| 82 | -23.765833 | -46.202914 | 0.817791 | 96.2292 | 0.001007 | 0.949389 | 0.000015 | 997 | 5.73989 | 0.000295 |
| 83 | -24.305974 | -48.43518 | 0.817364 | 98.2708 | 0.001007 | 0 | 0 | 619 | 5.56174 | 0.000328 |
| 84 | -24.098459 | -47.93892 | 0.81715 | 100 | 0.001007 | 0 | 0 | 855 | 3.6616 | 0.000315 |
| 85 | -24.090125 | -47.961701 | 0.817009 | 97.9931 | 0.001007 | 0 | 0 | 771 | 18.8547 | 0.000341 |
| 86 | -23.768004 | -46.171591 | 0.81505 | 96.2292 | 0.001007 | 0.949389 | 0.000075 | 775 | 12.1949 | 0.000301 |
| 87 | -24.057074 | -47.976788 | 0.814589 | 71.9931 | 0.001007 | 1.81682 | 0 | 806 | 3.02519 | 0.000347 |
| 88 | -24.099211 | -47.938848 | 0.81382 | 100 | 0.001007 | 0 | 0 | 854 | 4.53274 | 0.000315 |
| 89 | -23.750722 | -46.163446 | 0.813602 | 93.6389 | 0.001007 | 0.908408 | 0.000072 | 732 | 3.80862 | 0.000391 |
| 90 | -24.052656 | -47.967144 | 0.813052 | 74.9931 | 0.001007 | 0.040981 | 0 | 730 | 13.6704 | 0.000336 |
| 91 | -24.065893 | -47.938404 | 0.811264 | 94.7569 | 0.001007 | 0 | 0 | 852 | 16.9438 | 0.000344 |
| 92 | -24.063698 | -47.939532 | 0.809697 | 93.625 | 0.001007 | 0 | 0 | 836 | 16.3472 | 0.000338 |
| 93 | -24.279321 | -48.384477 | 0.808569 | 97.7292 | 0.001007 | 0.034151 | 0 | 792 | 35.057 | 0.000338 |
| 94 | -23.75106 | -46.157924 | 0.807976 | 93.7083 | 0.001007 | 0.949389 | 0.000081 | 730 | 1.43497 | 0.000377 |
| 95 | -24.285104 | -48.356431 | 0.806683 | 99.2361 | 0.001007 | 0 | 0 | 866 | 20.5713 | 0.000334 |
| 96 | -24.033415 | -47.950693 | 0.804392 | 66.4375 | 0.001007 | 0.245885 | 0 | 784 | 14.9938 | 0.000331 |
| 97 | -23.748738 | -46.157267 | 0.803371 | 93.7014 | 0.001007 | 0.785465 | 0.00008 | 734 | 4.30492 | 0.000382 |
| 98 | -24.06445 | -47.939459 | 0.803091 | 93.7431 | 0.001007 | 0 | 0 | 836 | 18.2166 | 0.000341 |
| 99 | -24.071723 | -47.957983 | 0.800625 | 93.0139 | 0.001007 | 0 | 0 | 831 | 14.4909 | 0.000325 |
| 100 | -24.060959 | -47.955359 | 0.799736 | 86.1736 | 0.001007 | 0 | 0 | 830 | 12.7292 | 0.000342 |
| 101 | -22.645953 | -45.429889 | 0.797789 | 80.8542 | 0.002002 | 0 | 0 | 1641 | 32.7035 | 0.000188 |
| 102 | -23.750791 | -46.164353 | 0.796764 | 93.5903 | 0.001007 | 0.908408 | 0.000068 | 727 | 4.33141 | 0.000393 |
| 103 | -24.042045 | -47.955353 | 0.79319 | 72.9931 | 0.001007 | 0.00683 | 0 | 770 | 18.131 | 0.000329 |
| 104 | -24.058823 | -47.957396 | 0.793077 | 84.3681 | 0.001007 | 0 | 0 | 830 | 13.089 | 0.000341 |
| 105 | -23.786052 | -46.219885 | 0.792415 | 97.1597 | 0.001007 | 0.949389 | 0.000017 | 895 | 16.6715 | 0.00026 |
| 106 | -24.071037 | -47.913188 | 0.791264 | 99.6389 | 0.001007 | 0 | 0 | 775 | 16.6482 | 0.000319 |
| 107 | -25.10827 | -47.961474 | 0.790385 | 81.051 | 0.000774 | 0 | 0 | 227 | 15.9218 | 0 |
| 108 | -24.075095 | -47.894484 | 0.789791 | 100 | 0.001007 | 0 | 0 | 775 | 7.12366 | 0.000308 |
| 109 | -24.076237 | -47.888879 | 0.789502 | 100 | 0.001007 | 0 | 0 | 791 | 9.31935 | 0.000305 |
| 110 | -24.073262 | -47.90107 | 0.788398 | 99.9583 | 0.001007 | 0 | 0 | 792 | 10.7213 | 0.000307 |
| 111 | -24.071999 | -47.904855 | 0.787146 | 99.7361 | 0.001007 | 0 | 0 | 792 | 15.3735 | 0.000315 |
| 112 | -24.072059 | -47.905765 | 0.783017 | 99.7361 | 0.001007 | 0 | 0 | 812 | 17.4625 | 0.000315 |
| 113 | -24.05416 | -47.966999 | 0.777346 | 76.3333 | 0.001007 | 0.040981 | 0 | 771 | 22.6071 | 0.000335 |
| 114 | -24.076539 | -47.893428 | 0.776675 | 100 | 0.001007 | 0 | 0 | 790 | 11.1255 | 0.000309 |
| 115 | -24.041985 | -47.954443 | 0.775597 | 73.4306 | 0.001007 | 0.00683 | 0 | 781 | 20.8387 | 0.000327 |
| 116 | -24.061171 | -47.9471 | 0.775148 | 89.5625 | 0.001007 | 0 | 0 | 775 | 16.7044 | 0.000348 |
| 117 | -24.131644 | -47.983343 | 0.774209 | 99.4583 | 0.001007 | 0.949389 | 0 | 866 | 39.114 | 0.000304 |
| 118 | -24.337909 | -48.366173 | 0.771618 | 100 | 0.001007 | 0 | 0 | 827 | 18.1335 | 0.000364 |
| 119 | -23.777649 | -46.208925 | 0.7696 | 97.1181 | 0.001007 | 0.949389 | 0.00002 | 921 | 18.9455 | 0.000275 |
| 120 | -24.073202 | -47.900161 | 0.76914 | 99.9583 | 0.001007 | 0 | 0 | 796 | 13.1117 | 0.000307 |
| 121 | -21.609574 | -47.624128 | 0.766949 | 17.5764 | 0.0016 | 58.2201 | 0.000089 | 739 | 6.7136 | 0.00018 |
| 122 | -23.77661 | -46.175211 | 0.765548 | 96.0833 | 0.001007 | 0.949389 | 0.000074 | 757 | 21.1374 | 0.000317 |
| 123 | -23.778331 | -46.207935 | 0.756785 | 97.1111 | 0.001007 | 0.949389 | 0.000021 | 881 | 22.211 | 0.000277 |
| 124 | -24.345701 | -48.453508 | 0.750685 | 98.5625 | 0.001007 | 0 | 0 | 439 | 19.9502 | 0.000329 |
| 125 | -24.313728 | -48.330829 | 0.75048 | 100 | 0.001007 | 0 | 0 | 927 | 12.7094 | 0.000382 |
| 126 | -23.772309 | -46.178429 | 0.74887 | 96.5556 | 0.001007 | 0.949389 | 0.000068 | 764 | 19.8109 | 0.000303 |
| 127 | -24.304179 | -48.442683 | 0.748246 | 97.0556 | 0.001007 | 0 | 0 | 716 | 23.2024 | 0.00034 |
| 128 | -24.055903 | -47.970493 | 0.744937 | 75.9375 | 0.001007 | 0.382488 | 0 | 794 | 32.007 | 0.000343 |
| 129 | -25.138053 | -47.989048 | 0.743567 | 90.7862 | 0.000774 | 0 | 0 | 502 | 46.3856 | 0.000004 |
| 130 | -24.058996 | -47.983011 | 0.74228 | 70.0208 | 0.001007 | 3.59948 | 0 | 814 | 9.3561 | 0.000355 |
| 131 | -23.799572 | -46.218395 | 0.738941 | 97.5139 | 0.001007 | 0.949389 | 0.00005 | 815 | 20.8189 | 0.000266 |
| 132 | -24.090789 | -47.948818 | 0.734773 | 99.3542 | 0.001007 | 0 | 0 | 830 | 32.7755 | 0.000327 |
| 133 | -24.046642 | -47.967724 | 0.733352 | 70.0486 | 0.001007 | 0.081962 | 0 | 748 | 26.0799 | 0.00034 |
| 134 | -24.057253 | -47.979517 | 0.731008 | 70.8472 | 0.001007 | 2.61594 | 0 | 812 | 12.3404 | 0.000349 |
| 135 | -23.803461 | -46.209736 | 0.725535 | 97.3889 | 0 | 0.949389 | 0.00008 | 769 | 13.8053 | 0.000276 |
| 136 | -24.070459 | -47.961767 | 0.724867 | 90.9931 | 0.001007 | 0 | 0 | 806 | 23.921 | 0.000324 |
| 137 | -25.138486 | -47.949301 | 0.722685 | 98.6319 | 0.000774 | 0 | 0 | 248 | 38.5715 | 0 |
| 138 | -23.814943 | -46.251453 | 0.718629 | 98.9931 | 0.001007 | 0.157093 | 0.000042 | 898 | 10.1626 | 0.000242 |
| 139 | -24.075031 | -47.870685 | 0.717689 | 100 | 0.001007 | 0 | 0 | 674 | 8.32065 | 0.000291 |
| 140 | -23.000153 | -46.066941 | 0.713772 | 73.2986 | 0.002002 | 0 | 0 | 785 | 30.4599 | 0.000369 |
| 141 | -24.075783 | -47.870612 | 0.711708 | 100 | 0.001007 | 0 | 0 | 663 | 8.84318 | 0.000291 |
| 142 | -24.158049 | -47.970722 | 0.709122 | 100 | 0.001007 | 0 | 0 | 697 | 9.6142 | 0.000271 |
| 143 | -24.021601 | -47.920718 | 0.704853 | 65.3403 | 0.001007 | 8.51718 | 0 | 813 | 7.96091 | 0.000324 |
| 144 | -22.44999 | -44.883038 | 0.700544 | 91.3333 | 0.002002 | 0 | 0 | 1666 | 64.2897 | 0.000183 |
| 145 | -21.616855 | -47.807517 | 0.699889 | 60.1944 | 0.0016 | 0 | 0 | 532 | 1.97218 | 0.000273 |
| 146 | -24.329751 | -48.58522 | 0.690383 | 94.75 | 0.001007 | 0 | 0.000065 | 879 | 6.54798 | 0.00035 |
| 147 | -24.07762 | -47.886914 | 0.689598 | 100 | 0.001007 | 0 | 0 | 803 | 23.4818 | 0.000308 |
| 148 | -21.624713 | -47.80048 | 0.688847 | 56.8819 | 0.0016 | 0 | 0 | 543 | 1.43497 | 0.000278 |
| 149 | -23.742723 | -46.147878 | 0.688405 | 95.2569 | 0.001007 | 0.027321 | 0.000085 | 738 | 1.76397 | 0.000416 |
| 150 | -25.107186 | -47.968041 | 0.686749 | 78.5724 | 0.000774 | 0 | 0 | 297 | 44.0147 | 0 |
| 151 | -24.107348 | -48.039711 | 0.683796 | 79.2778 | 0.001007 | 17.1163 | 0 | 871 | 5.64747 | 0.000333 |
| 152 | -24.327552 | -48.586339 | 0.681214 | 94.3194 | 0.001007 | 0 | 0.000066 | 878 | 9.62847 | 0.000345 |
| 153 | -24.297246 | -48.416734 | 0.676294 | 99.4167 | 0.001007 | 0.034151 | 0 | 640 | 35.2983 | 0.000301 |
| 154 | -23.769024 | -46.165079 | 0.672272 | 95.9444 | 0.001007 | 0.949389 | 0.000085 | 740 | 25.6928 | 0.000297 |
| 155 | -24.020398 | -47.92541 | 0.671545 | 63.2361 | 0.001007 | 7.86832 | 0 | 746 | 10.4905 | 0.000331 |
| 156 | -23.811321 | -46.233561 | 0.669932 | 98.7708 | 0.001007 | 0.949389 | 0.000063 | 891 | 27.4151 | 0.000278 |
| 157 | -23.744294 | -46.148618 | 0.667502 | 95.3264 | 0.001007 | 0.027321 | 0.000085 | 747 | 19.0491 | 0.000402 |
| 158 | -24.023345 | -47.92421 | 0.664484 | 66.7708 | 0.001007 | 7.27409 | 0 | 769 | 12.3422 | 0.000334 |
| 159 | -24.051607 | -47.871129 | 0.660593 | 98.6111 | 0.001007 | 1.23625 | 0 | 750 | 36.0804 | 0.000297 |
| 160 | -24.058124 | -47.981264 | 0.659649 | 69.6875 | 0.001007 | 3.29895 | 0 | 825 | 0.771275 | 0.000353 |
| 161 | -24.340269 | -48.439335 | 0.656991 | 99.3194 | 0.001007 | 0 | 0 | 442 | 30.0462 | 0.00032 |
| 162 | -21.603574 | -47.768381 | 0.656725 | 78.7292 | 0.0016 | 0.170753 | 0.000043 | 596 | 0.956649 | 0.000224 |
| 163 | -24.328013 | -48.42488 | 0.655663 | 99.4861 | 0.001007 | 0 | 0 | 607 | 27.5707 | 0.00032 |
| 164 | -22.316733 | -48.966945 | 0.651917 | 1.23611 | 0 | 60.112 | 0.0001 | 556 | 2.78909 | 0.00021 |
| 165 | -25.107674 | -47.929221 | 0.650628 | 85.7793 | 0.000774 | 0 | 0 | 206 | 32.6328 | 0 |
| 166 | -24.060559 | -47.983776 | 0.647219 | 70.9931 | 0.001007 | 3.57899 | 0 | 817 | 20.3937 | 0.00036 |
| 167 | -21.657074 | -47.708451 | 0.64668 | 48.7014 | 0.0016 | 8.12103 | 0 | 624 | 2.43899 | 0.000213 |
| 168 | -24.080085 | -47.878434 | 0.644403 | 100 | 0.001007 | 0 | 0 | 729 | 25.3512 | 0.000305 |
| 169 | -23.742991 | -46.14145 | 0.643036 | 95.8958 | 0.001007 | 0 | 0.000088 | 738 | 11.1748 | 0.000407 |
| 170 | -24.311647 | -48.41723 | 0.632821 | 99.9097 | 0.001007 | 0 | 0 | 631 | 25.6919 | 0.000317 |
| 171 | -23.741696 | -46.144336 | 0.629679 | 95.6736 | 0.001007 | 0 | 0.000086 | 739 | 11.2299 | 0.000414 |
| 172 | -23.745114 | -46.149442 | 0.628506 | 95.1458 | 0.001007 | 0.061471 | 0.000086 | 754 | 22.8196 | 0.000398 |
| 173 | -24.318181 | -48.424876 | 0.628436 | 99.2986 | 0.001007 | 0 | 0 | 560 | 31.2343 | 0.000315 |
| 174 | -24.322865 | -48.427193 | 0.619294 | 99.3194 | 0.001007 | 0 | 0 | 545 | 35.4063 | 0.000321 |
| 175 | -22.516095 | -51.049968 | 0.612175 | 26.5347 | 0 | 0.280035 | 0.000074 | 456 | 3.56023 | 0.000128 |
| 176 | -24.232828 | -49.11729 | 0.609185 | 16.5069 | 0 | 60.8633 | 0 | 1072 | 0.956649 | 0.000394 |
| 177 | -23.788648 | -46.22417 | 0.599457 | 97.1111 | 0.001007 | 0.949389 | 0.000015 | 948 | 34.3975 | 0.000254 |
| 178 | -21.591275 | -47.799189 | 0.587675 | 80.0417 | 0.0016 | 0 | 0.000044 | 548 | 9.32671 | 0.000255 |
| 179 | -21.60641 | -47.821087 | 0.587059 | 65.1389 | 0.0016 | 0 | 0.000019 | 524 | 8.80169 | 0.000259 |
| 180 | -21.596851 | -47.803145 | 0.581996 | 75.3889 | 0.0016 | 0 | 0.000039 | 590 | 7.59618 | 0.00026 |
| 181 | -22.812649 | -48.908208 | 0.58051 | 16.3611 | 0.001322 | 55.5768 | 0 | 683 | 5.65152 | 0.000171 |
| 182 | -22.774871 | -48.958622 | 0.577697 | 16.9028 | 0 | 61.6829 | 0 | 649 | 3.54089 | 0.000224 |
| 183 | -22.240423 | -47.807141 | 0.577374 | 8.06944 | 0 | 40.974 | 0.000114 | 771 | 3.48225 | 0.000201 |
| 184 | -22.776816 | -48.965697 | 0.574587 | 18.6806 | 0 | 61.4644 | 0 | 648 | 3.34143 | 0.000226 |
| 185 | -23.28952 | -45.056312 | 0.573905 | 99.9167 | 0.001007 | 0 | 0 | 1005 | 28.9921 | 0.000301 |
| 186 | -21.59504 | -47.798829 | 0.573674 | 77.7292 | 0.0016 | 0 | 0.000042 | 550 | 10.6571 | 0.000255 |
| 187 | -21.609178 | -47.64033 | 0.573615 | 20.0972 | 0 | 49.8395 | 0.000093 | 686 | 5.20473 | 0.000168 |
| 188 | -23.813031 | -46.256236 | 0.570954 | 99.0208 | 0.001007 | 0 | 0.000044 | 835 | 15.883 | 0.000243 |
| 189 | -24.319285 | -48.394509 | 0.552557 | 100 | 0.001007 | 0 | 0 | 732 | 43.0943 | 0.000317 |
| 190 | -24.308928 | -48.422068 | 0.549471 | 99.4167 | 0.001007 | 0 | 0 | 633 | 38.8174 | 0.000314 |
| 191 | -24.307075 | -48.428659 | 0.544877 | 98.6319 | 0.001007 | 0 | 0 | 623 | 47.5196 | 0.000319 |
| 192 | -21.983445 | -46.633659 | 0.542427 | 31.3264 | 0.003499 | 0.443959 | 0.000004 | 1407 | 18.3442 | 0.0001 |
| 193 | -22.46682 | -51.216823 | 0.539132 | 15.7431 | 0 | 0 | 0 | 417 | 3.32083 | 0.000189 |
| 194 | -20.533071 | -49.199956 | 0.536914 | 27.9306 | 0.001218 | 31.5347 | 0 | 450 | 0.956649 | 0.000225 |
| 195 | -25.169355 | -47.970308 | 0.53438 | 93.4779 | 0.000774 | 0 | 0 | 114 | 36.1722 | 0 |
| 196 | -22.449612 | -51.282993 | 0.532827 | 18.4028 | 0 | 0 | 0 | 424 | 3.34143 | 0.000193 |
| 197 | -24.310198 | -48.430203 | 0.532817 | 98.8889 | 0.001007 | 0 | 0 | 595 | 48.4243 | 0.000321 |
| 198 | -22.216613 | -47.76883 | 0.527928 | 6.47917 | 0 | 37.7706 | 0.000132 | 815 | 5.17387 | 0.000165 |
| 199 | -22.400017 | -52.483217 | 0.524633 | 12.6319 | 0 | 0 | 0.000076 | 408 | 2.78087 | 0.000118 |
| 200 | -21.350098 | -49.349483 | 0.523366 | 11.3681 | 0 | 0.409808 | 0.000065 | 381 | 4.99385 | 0.000353 |
| 201 | -20.466531 | -48.799946 | 0.519648 | 15.4792 | 0 | 3.84537 | 0 | 497 | 3.94436 | 0.000391 |
| 202 | -20.5006 | -50.166782 | 0.517256 | 10.0208 | 0 | 0 | 0 | 382 | 1.15196 | 0.000337 |
| 203 | -21.532896 | -50.816013 | 0.516684 | 8.97917 | 0 | 22.5941 | 0 | 350 | 3.73583 | 0.000352 |
| 204 | -23.791652 | -46.223839 | 0.50468 | 97.0417 | 0.001007 | 0.949389 | 0.000018 | 945 | 47.1978 | 0.000257 |
| 205 | -21.039549 | -50.866514 | 0.503793 | 10.3333 | 0 | 0 | 0 | 350 | 2.68033 | 0.000198 |
| 206 | -24.328247 | -48.585358 | 0.494067 | 94.4931 | 0.001007 | 0 | 0.000065 | 891 | 28.9724 | 0.000346 |
| 207 | -21.578718 | -48.062506 | 0.492501 | 14.2431 | 0 | 0 | 0 | 537 | 7.37613 | 0.00038 |
| 208 | -21.516749 | -50.766996 | 0.489613 | 7.0625 | 0 | 9.84905 | 0 | 372 | 3.42929 | 0.000323 |
| 209 | -20.937323 | -48.90618 | 0.488734 | 47.7569 | 0.002145 | 0 | 0.000025 | 501 | 4.69148 | 0.00027 |
| 210 | -23.745038 | -46.138481 | 0.48426 | 96.1042 | 0.001007 | 0 | 0.000094 | 765 | 26.0606 | 0.000373 |
| 211 | -22.450048 | -52.450482 | 0.483712 | 19.6319 | 0 | 0 | 0.000059 | 429 | 3.56023 | 0.000127 |
| 212 | -22.65506 | -47.156821 | 0.480912 | 7.40972 | 0 | 0.01366 | 0 | 554 | 9.86091 | 0.000261 |
| 213 | -22.024645 | -48.545694 | 0.48063 | 31.0417 | 0.002156 | 47.2645 | 0 | 430 | 2.01805 | 0.000202 |
| 214 | -22.304032 | -48.021304 | 0.479092 | 10.0208 | 0 | 44.0202 | 0.000066 | 756 | 3.25823 | 0.000242 |
| 215 | -23.770601 | -46.175875 | 0.476847 | 96.4792 | 0.001007 | 0.949389 | 0.00007 | 798 | 54.0773 | 0.000306 |
| 216 | -21.653647 | -51.531088 | 0.474081 | 10.0486 | 0 | 0 | 0 | 350 | 2.73942 | 0.000242 |
| 217 | -21.016649 | -47.333647 | 0.471078 | 33.3542 | 0 | 0.799126 | 0.000094 | 884 | 0.956649 | 0.000235 |
| 218 | -22.254455 | -47.813012 | 0.471012 | 11.3472 | 0 | 32.9144 | 0.000109 | 762 | 1.54255 | 0.000206 |
| 219 | -24.367086 | -48.480884 | 0.4705 | 99.9306 | 0.001007 | 0 | 0 | 231 | 10.749 | 0.000343 |
| 220 | -21.627742 | -47.645716 | 0.466089 | 33.0625 | 0 | 34.5468 | 0.000072 | 657 | 4.54786 | 0.000154 |
| 221 | -22.566732 | -45.183521 | 0.458853 | 77.9444 | 0.002002 | 0 | 0.000066 | 1020 | 21.1168 | 0.000275 |
| 222 | -20.116222 | -47.599493 | 0.452844 | 31.5069 | 0.001858 | 0 | 0.000088 | 642 | 14.885 | 0.000305 |
| 223 | -22.838196 | -48.88059 | 0.451938 | 37.6944 | 0 | 25.6335 | 0 | 737 | 6.82514 | 0.000166 |
| 224 | -22.750266 | -48.816794 | 0.45017 | 15.0833 | 0 | 5.70316 | 0 | 676 | 3.58584 | 0.000206 |
| 225 | -21.629506 | -47.583571 | 0.449066 | 17.8681 | 0 | 36.712 | 0.000061 | 678 | 2.18149 | 0.000188 |
| 226 | -20.549736 | -50.150578 | 0.448799 | 6.29167 | 0 | 0.389318 | 0.000015 | 483 | 7.63523 | 0.00033 |
| 227 | -21.577909 | -47.736728 | 0.445076 | 58.5278 | 0.0016 | 5.07479 | 0.000058 | 636 | 12.9006 | 0.000195 |
| 228 | -23.957344 | -47.242609 | 0.443241 | 87.0486 | 0.001007 | 0 | 0.000004 | 783 | 24.5022 | 0.000678 |
| 229 | -24.399226 | -48.571511 | 0.441661 | 100 | 0.001007 | 0 | 0.000041 | 672 | 23.1383 | 0.000379 |
| 230 | -22.245309 | -47.711914 | 0.439112 | 12.9722 | 0 | 9.10457 | 0.00012 | 840 | 3.93275 | 0.000188 |
| 231 | -22.232626 | -47.893611 | 0.437361 | 2.8125 | 0 | 36.1041 | 0.000086 | 735 | 0.907557 | 0.000287 |
| 232 | -22.286085 | -47.698934 | 0.436076 | 13.9444 | 0 | 1.24992 | 0.000072 | 701 | 8.6125 | 0.000139 |
| 233 | -21.549747 | -50.717107 | 0.430402 | 4.46528 | 0 | 14.6711 | 0 | 409 | 2.87791 | 0.0003 |
| 234 | -21.916881 | -47.250316 | 0.42729 | 12.4514 | 0 | 0 | 0 | 612 | 5.39892 | 0.000278 |
| 235 | -20.216781 | -47.283325 | 0.424364 | 35.2292 | 0 | 0 | 0 | 701 | 7.1108 | 0.000523 |
| 236 | -22.816425 | -48.883414 | 0.424036 | 32.4792 | 0 | 41.1106 | 0 | 713 | 6.12927 | 0.000156 |
| 237 | -22.499854 | -48.916643 | 0.417586 | 0.881944 | 0 | 75.6642 | 0.000065 | 597 | 8.26547 | 0.000249 |
| 238 | -22.11661 | -48.199431 | 0.417501 | 24.8472 | 0 | 0.826446 | 0.000062 | 605 | 5.45374 | 0.000228 |
| 239 | -24.308872 | -48.409234 | 0.416469 | 99.8819 | 0.001007 | 0 | 0 | 602 | 53.2571 | 0.000309 |
| 240 | -22.600553 | -49.016349 | 0.415637 | 20.3472 | 0 | 6.21542 | 0 | 713 | 1.55731 | 0.000252 |
| 241 | -22.62965 | -47.140388 | 0.412899 | 4.39583 | 0 | 0 | 0 | 559 | 4.03611 | 0.000275 |
| 242 | -20.199759 | -47.300104 | 0.411965 | 24.8958 | 0 | 0 | 0 | 760 | 9.26024 | 0.000495 |
| 243 | -22.631218 | -47.141134 | 0.411394 | 4.38195 | 0 | 0 | 0 | 557 | 4.28361 | 0.000274 |
| 244 | -22.641619 | -47.138265 | 0.408399 | 5.15972 | 0 | 0 | 0 | 548 | 4.40474 | 0.000271 |
| 245 | -22.641747 | -47.140063 | 0.405823 | 5.15278 | 0 | 0 | 0 | 549 | 4.5125 | 0.000269 |
| 246 | -22.643251 | -47.13991 | 0.401881 | 5.34722 | 0 | 0 | 0 | 549 | 5.46631 | 0.000269 |
| 247 | -22.634418 | -47.143525 | 0.401334 | 4.375 | 0 | 0 | 0 | 557 | 5.95893 | 0.00027 |
| 248 | -22.050161 | -48.133533 | 0.40068 | 18.8889 | 0 | 1.09282 | 0.000063 | 661 | 4.92 | 0.000258 |
| 249 | -22.221307 | -47.905518 | 0.396429 | 1.79167 | 0 | 34.4239 | 0.000078 | 727 | 3.48225 | 0.000265 |
| 250 | -22.632034 | -47.141957 | 0.395737 | 4.38195 | 0 | 0 | 0 | 559 | 7.05589 | 0.000273 |
| 251 | -22.467019 | -48.89964 | 0.392648 | 0 | 0 | 89.5021 | 0.000061 | 572 | 6.82514 | 0.000276 |
| 252 | -22.334823 | -47.4937 | 0.389467 | 6.63195 | 0 | 0.245885 | 0 | 601 | 0.771275 | 0.000266 |
| 253 | -21.499917 | -49.283201 | 0.386184 | 4.72917 | 0 | 0.061471 | 0.000052 | 436 | 3.56023 | 0.000415 |
| 254 | -22.63109 | -47.139336 | 0.38403 | 4.41667 | 0 | 0 | 0 | 563 | 7.32321 | 0.000274 |
| 255 | -21.050192 | -47.400082 | 0.377204 | 24.3125 | 0 | 4.58302 | 0.000075 | 760 | 5.87386 | 0.000202 |
| 256 | -21.170119 | -47.294294 | 0.374807 | 25.7639 | 0 | 0 | 0.000066 | 740 | 6.90512 | 0.00032 |
| 257 | -21.999572 | -48.449814 | 0.372691 | 9.52083 | 0 | 13.0251 | 0.000067 | 536 | 4.45124 | 0.000165 |
| 258 | -21.574359 | -51.6636 | 0.369869 | 8.67361 | 0 | 0 | 0.000064 | 350 | 5.13837 | 0.000252 |
| 259 | -22.630466 | -47.141211 | 0.369307 | 4.375 | 0 | 0 | 0 | 570 | 8.9384 | 0.000273 |
| 260 | -22.083139 | -48.548528 | 0.369089 | 29 | 0 | 9.4734 | 0.000003 | 472 | 5.59864 | 0.00028 |
| 261 | -20.833214 | -48.567256 | 0.367499 | 11.2292 | 0 | 0 | 0.000071 | 547 | 5.28327 | 0.000324 |
| 262 | -21.77225 | -47.908618 | 0.364826 | 12.0556 | 0 | 0 | 0 | 711 | 11.9734 | 0.00021 |
| 263 | -22.634226 | -47.140828 | 0.364775 | 4.33333 | 0 | 0 | 0 | 563 | 8.19039 | 0.000272 |
| 264 | -21.317062 | -49.316425 | 0.362629 | 6.54861 | 0 | 1.82365 | 0.000072 | 428 | 8.01818 | 0.00031 |
| 265 | -22.629266 | -47.134995 | 0.36215 | 4.57639 | 0 | 0 | 0 | 589 | 9.06548 | 0.000276 |
| 266 | -22.372386 | -50.973522 | 0.361424 | 18.8958 | 0 | 0 | 0.000103 | 401 | 5.45374 | 0.000198 |
| 267 | -22.431419 | -47.521217 | 0.360328 | 0.597222 | 0 | 18.9604 | 0.000114 | 731 | 3.2087 | 0.000316 |
| 268 | -23.667027 | -47.032985 | 0.358142 | 35.5972 | 0 | 0 | 0.000081 | 923 | 6.05416 | 0.000403 |
| 269 | -21.148416 | -49.461908 | 0.352165 | 0.604167 | 0 | 0 | 0 | 413 | 7.19079 | 0.000442 |
| 270 | -20.100007 | -47.649863 | 0.352138 | 28.6944 | 0 | 0 | 0.000114 | 789 | 10.4292 | 0.000337 |
| 271 | -22.62845 | -47.134172 | 0.351777 | 4.63889 | 0 | 0 | 0 | 589 | 9.79807 | 0.000275 |
| 272 | -22.660061 | -47.195257 | 0.348755 | 9.61111 | 0 | 0 | 0 | 540 | 8.90249 | 0.00049 |
| 273 | -22.183117 | -52.199882 | 0.34617 | 0.770833 | 0 | 0 | 0.000093 | 339 | 5.14282 | 0.000172 |
| 274 | -22.63973 | -47.133023 | 0.345226 | 4.79167 | 0 | 0 | 0 | 561 | 12.1178 | 0.000275 |
| 275 | -22.649604 | -48.117089 | 0.343167 | 15.8889 | 0 | 16.2284 | 0.000061 | 459 | 1.54255 | 0.000382 |
| 276 | -22.413245 | -47.510356 | 0.33121 | 0.659722 | 0 | 20.5109 | 0.000095 | 659 | 4.1147 | 0.000296 |
| 277 | -21.38318 | -49.383449 | 0.327183 | 13.6875 | 0 | 0 | 0.000059 | 389 | 8.03813 | 0.000414 |
| 278 | -20.416535 | -49.216828 | 0.326289 | 22.7569 | 0.003327 | 0 | 0.000102 | 449 | 8.7286 | 0.000232 |
| 279 | -22.636354 | -47.1388 | 0.324308 | 4.23611 | 0 | 0 | 0 | 573 | 10.6807 | 0.000272 |
| 280 | -23.068887 | -45.93658 | 0.323519 | 39.6667 | 0.003736 | 0 | 0.00006 | 726 | 16.0335 | 0.000361 |
| 281 | -21.181013 | -49.146233 | 0.322283 | 1 | 0 | 0 | 0 | 448 | 6.42096 | 0.000225 |
| 282 | -21.641726 | -47.650648 | 0.3221 | 37.7222 | 0 | 24.9095 | 0.000059 | 641 | 6.92828 | 0.000156 |
| 283 | -22.133432 | -48.399781 | 0.321476 | 22.875 | 0 | 0 | 0.0001 | 464 | 4.70609 | 0.000387 |
| 284 | -21.805942 | -46.941149 | 0.321078 | 13.5833 | 0 | 0 | 0.000121 | 735 | 3.79055 | 0.000241 |
| 285 | -21.175828 | -48.125133 | 0.319095 | 16.5347 | 0 | 0 | 0.000066 | 503 | 5.84261 | 0.000206 |
| 286 | -24.233171 | -49.199714 | 0.31619 | 0.055556 | 0 | 85.0625 | 0 | 1033 | 9.31935 | 0.000372 |
| 287 | -21.499683 | -49.317287 | 0.310864 | 5.82639 | 0 | 1.9944 | 0.000007 | 444 | 0.605038 | 0.000399 |
| 288 | -21.654825 | -47.609852 | 0.305583 | 23.8958 | 0 | 19.0766 | 0.000064 | 715 | 5.22229 | 0.000224 |
| 289 | -22.290271 | -48.099327 | 0.304415 | 10.9583 | 0 | 16.9046 | 0.000066 | 678 | 4.1147 | 0.000238 |
| 290 | -22.627505 | -47.131552 | 0.303106 | 4.77083 | 0.005057 | 0 | 0 | 610 | 3.87413 | 0.000279 |
| 291 | -22.499946 | -47.983772 | 0.302648 | 19.2847 | 0 | 0 | 0.000076 | 913 | 4.27826 | 0.000243 |
| 292 | -22.300511 | -51.033626 | 0.299902 | 8.34028 | 0 | 0 | 0.000052 | 487 | 4.1147 | 0.00018 |
| 293 | -21.929617 | -47.19501 | 0.293702 | 15.3333 | 0 | 0 | 0.000031 | 633 | 6.43164 | 0.000295 |
| 294 | -22.414116 | -47.766019 | 0.287521 | 19.7917 | 0.002847 | 0 | 0.00008 | 647 | 18.4412 | 0.000207 |
| 295 | -21.264126 | -47.859949 | 0.287374 | 7.21528 | 0 | 0 | 0.000089 | 689 | 8.99453 | 0.000175 |
| 296 | -21.19907 | -48.166827 | 0.28672 | 10.3681 | 0 | 0 | 0.000067 | 502 | 4.8071 | 0.000238 |
| 297 | -21.927678 | -46.93675 | 0.286137 | 15.6806 | 0 | 0 | 0.000033 | 683 | 8.47053 | 0.000302 |
| 298 | -21.243402 | -47.570011 | 0.281628 | 11.6597 | 0 | 0 | 0.000075 | 659 | 8.99708 | 0.000284 |
| 299 | -22.352258 | -47.915596 | 0.281529 | 16.9028 | 0 | 1.78949 | 0.000092 | 935 | 2.13913 | 0.000258 |
| 300 | -20.13311 | -47.667118 | 0.277315 | 43.9028 | 0.001858 | 0 | 0.000151 | 576 | 9.41461 | 0.000291 |
| 301 | -20.133666 | -47.566746 | 0.275947 | 31.3403 | 0.001858 | 0 | 0.000103 | 849 | 33.7644 | 0.000248 |
| 302 | -22.099946 | -48.016223 | 0.27368 | 26.5486 | 0 | 4.61034 | 0.00006 | 684 | 6.3204 | 0.000228 |
| 303 | -21.286814 | -48.278704 | 0.270161 | 5.13889 | 0 | 0 | 0.000113 | 615 | 3.52795 | 0.000294 |
| 304 | -22.540188 | -47.593713 | 0.264869 | 3.375 | 0 | 0 | 0.000064 | 644 | 3.48225 | 0.000339 |
| 305 | -22.012199 | -46.952391 | 0.262706 | 12.9375 | 0 | 0 | 0.000102 | 652 | 8.05235 | 0.000308 |
| 306 | -21.485695 | -47.641521 | 0.261216 | 19.4167 | 0 | 3.03258 | 0.000131 | 661 | 8.65225 | 0.000198 |
| 307 | -22.418405 | -47.784582 | 0.25932 | 23.5347 | 0 | 0 | 0.000067 | 703 | 10.1985 | 0.000231 |
| 308 | -22.04093 | -47.637077 | 0.253275 | 14.4514 | 0 | 0 | 0.000084 | 1007 | 6.4174 | 0.000168 |
| 309 | -22.886152 | -48.909904 | 0.246586 | 10.0556 | 0 | 5.22505 | 0.000047 | 672 | 7.92634 | 0.000264 |
| 310 | -22.53326 | -50.200579 | 0.24574 | 9.44444 | 0 | 0 | 0.000041 | 546 | 7.78656 | 0.000216 |
| 311 | -21.499916 | -50.866762 | 0.245267 | 2.9375 | 0 | 29.9911 | 0 | 335 | 9.86091 | 0.000289 |
| 312 | -22.059803 | -47.493747 | 0.245134 | 6.75 | 0 | 0 | 0.000112 | 786 | 2.18149 | 0.000275 |
| 313 | -22.649456 | -48.184083 | 0.23666 | 19.5903 | 0 | 14.8965 | 0.000067 | 558 | 4.30492 | 0.000539 |
| 314 | -21.585758 | -48.4469 | 0.235689 | 10.8403 | 0.007201 | 0 | 0.000064 | 518 | 3.64281 | 0.000186 |
| 315 | -22.499787 | -48.049789 | 0.23566 | 16.8056 | 0 | 0 | 0.000044 | 897 | 10.5014 | 0.000237 |
| 316 | -24.197748 | -47.930235 | 0.2321 | 91.1319 | 0.001007 | 0 | 0 | 61 | 5.01215 | 0.000247 |
| 317 | -24.203911 | -47.943386 | 0.228408 | 91.9861 | 0.001007 | 0 | 0 | 43 | 3.02519 | 0.000261 |
| 318 | -25.106749 | -48.007779 | 0.221494 | 77.3611 | 0.000774 | 0 | 0 | 10 | 12.3181 | 0.00004 |
| 319 | -22.225018 | -48.270534 | 0.220716 | 13.5694 | 0.009084 | 1.41384 | 0.000065 | 459 | 0.605038 | 0.000319 |
| 320 | -22.575071 | -47.561334 | 0.218613 | 2.97222 | 0 | 0 | 0.00012 | 594 | 2.43899 | 0.000286 |
| 321 | -22.170251 | -47.426031 | 0.20515 | 1.80556 | 0 | 2.03538 | 0.000063 | 634 | 4.53274 | 0.000216 |
| 322 | -25.168858 | -48.009148 | 0.201848 | 92.0779 | 0.000774 | 0 | 0 | 13 | 1.30118 | 0.000049 |
| 323 | -24.133331 | -48.88336 | 0.195445 | 44.8264 | 0 | 8.34642 | 0.000054 | 705 | 16.7071 | 0.000301 |
| 324 | -20.765116 | -47.860724 | 0.194867 | 8.81944 | 0 | 0 | 0.000126 | 717 | 2.20237 | 0.000224 |
| 325 | -21.213642 | -47.84236 | 0.191324 | 6.81945 | 0.004237 | 0 | 0.000136 | 585 | 8.46242 | 0.000191 |
| 326 | -22.308322 | -47.836724 | 0.190134 | 18.7986 | 0 | 19.459 | 0.000087 | 679 | 14.3194 | 0.000256 |
| 327 | -22.195445 | -47.636454 | 0.18989 | 20.5278 | 0 | 0 | 0.000061 | 617 | 17.0809 | 0.000237 |
| 328 | -22.350392 | -49.916089 | 0.178785 | 13.8472 | 0 | 0 | 0.000072 | 506 | 28.0235 | 0.000196 |
| 329 | -21.790922 | -46.787945 | 0.172192 | 16.9861 | 0 | 0 | 0.000056 | 1015 | 2.57586 | 0.000236 |
| 330 | -22.907521 | -48.925251 | 0.171396 | 2.97222 | 0 | 3.73608 | 0.000059 | 626 | 11.2299 | 0.00029 |
| 331 | -21.142248 | -49.008251 | 0.169537 | 0.597222 | 0 | 0 | 0.000132 | 515 | 6.54798 | 0.000229 |
| 332 | -22.885677 | -48.951644 | 0.168427 | 3.4375 | 0 | 9.66464 | 0.000054 | 660 | 8.7286 | 0.000253 |
| 333 | -22.194555 | -47.689767 | 0.163189 | 25.9722 | 0 | 4.38495 | 0.000123 | 726 | 24.1447 | 0.000189 |
| 334 | -21.5335 | -49.30011 | 0.162928 | 6.875 | 0.005276 | 1.73485 | 0.000009 | 393 | 7.27305 | 0.000597 |
| 335 | -21.736706 | -48.170823 | 0.16037 | 1.86111 | 0 | 0 | 0.000096 | 666 | 9.45583 | 0.000241 |
| 336 | -21.021779 | -49.021257 | 0.117697 | 2.32639 | 0 | 0 | 0.000146 | 525 | 3.44925 | 0.000233 |
| 337 | -21.203029 | -49.604977 | 0.095608 | 1.19444 | 0 | 0 | 0.000065 | 478 | 5.79148 | 0.000698 |
| 338 | -23.77307 | -46.098803 | 0.083752 | 89.892 | 0.001007 | 0 | 0.000114 | 37 | 12.3181 | 0.000227 |
| 339 | -21.01825 | -49.136785 | 0.072342 | 1.95139 | 0 | 0 | 0.000156 | 497 | 11.8331 | 0.000213 |
| 340 | -25.1368 | -48.027951 | 0.043101 | 89.5417 | 0 | 0 | 0 | 2 | 0.213913 | 0.000077 |
| 341 | -20.10003 | -47.349849 | 0 | 0 | 0 | 0 | 0 | 0 | 0 | 0 |
| 342 | -21.933088 | -46.649764 | 0 | 0 | 0 | 0 | 0 | 0 | 0 | 0 |
