# Supplementary material for: Using Species Distribution Models to Predict Potential Landscape Restoration Effects on Puma Conservation
Source: PLoS One. 2016 Jan 6;11(1):e0145232. doi: 10.1371/journal.pone.0145232 (PMC4703218; doi:10.1371/journal.pone.0145232)
Supplement: S2 Table — (DOCX) [file pone.0145232.s005.docx]

Table S2. Ecological relationships relevant for pumas published in the literature that were considered as potential explanatory variables (n = 13) to guide the species distribution modelling (SDM).

| Potential explanatory variables | Ecological relationships considered to be relevant for pumas | References |
| --- | --- | --- |
| 1. percentage of native vegetation; 2. distance to native vegetation | The puma is believed to prefer habitats containing proportionally more native vegetation cover | LaRue & Nielsen 2011 |
| (3) edge density of native vegetation patches | Edge density of native vegetation patches was reported as negatively affecting puma’s density | Lyra-Jorge et al*.* 2010 |
| 1. percentage of exotic forest crops; 2. distance to exotic forest crops | Pumas have been reported using this land use type as a complementary habitat | Mazzolli 2010 |
| (6) distance to protected areas | Pumas were reported to prefer areas less disturbed such as protected areas | Paviollo et al. 2009 |
| 1. watercourse density; 2. distance to watercourses | Pumas positively respond to availability of surface water | De Angelo *et al.* 2011; Sollmann *et al.* 2012 |
| 1. distance to roads; 2. road density | The dispersal capacity of pumas is affected by paved roads | Dickson *et al.* 2005; Dickson *et al.* 2013 |
| 1. elevation; 2. slope | The dispersal capacity of pumas is affected by topography | Dickson and Beier 2007 |
| (13) distance to urban areas | Pumas respond negatively to proximity to human settlements | De Angelo *et al.* 2011; Sollmann *et al.* 2012 |
