# Supplementary material for: Using Species Distribution Models to Predict Potential Landscape Restoration Effects on Puma Conservation
Source: PLoS One. 2016 Jan 6;11(1):e0145232. doi: 10.1371/journal.pone.0145232 (PMC4703218; doi:10.1371/journal.pone.0145232)
Supplement: S3 Table — (DOCX) [file pone.0145232.s006.docx]

Table S3. Pearson’s correlation analysis of the environmental variables.

| Variable | 1 | 2 | 3 | 4 | 5 | 6 | 7 | 8 | 9 | 10 | 11 | 12 | 13 | 14 | 15 |
| --- | --- | --- | --- | --- | --- | --- | --- | --- | --- | --- | --- | --- | --- | --- | --- |
| 1. Percentage of vegetation | 1.00 |  |  |  |  |  |  |  |  |  |  |  |  |  |  |
| 2. Distance to natural vegetation | **-0.56** | 1.00 |  |  |  |  |  |  |  |  |  |  |  |  |  |
| 3. Percentage of exotic forest | -0.36 | **0.58** | 1.00 |  |  |  |  |  |  |  |  |  |  |  |  |
| 4. Distance to exotic forest | -0.06 | -0.08 | -0.30 | 1.00 |  |  |  |  |  |  |  |  |  |  |  |
| 5. Number of natural vegetation patches | **-0.55** | 0.08 | 0.02 | 0.10 | 1.00 |  |  |  |  |  |  |  |  |  |  |
| 6. Edge density of natural vegetation | 0.26 | -0.34 | -0.17 | -0.03 | -0.08 | 1.00 |  |  |  |  |  |  |  |  |  |
| 7. Distance to strictly protected areas | **-0.73** | 0.39 | 0.10 | 0.11 | 0.50 | -0.16 | 1.00 |  |  |  |  |  |  |  |  |
| 8. Density of streams | 0.18 | -0.06 | -0.14 | -0.34 | -0.13 | 0.13 | -0.05 | 1.00 |  |  |  |  |  |  |  |
| 9. Distance to streams | 0.02 | 0.01 | 0.01 | 0.48 | -0.04 | -0.13 | -0.05 | **-0.54** | 1.00 |  |  |  |  |  |  |
| 10. Human population density | -0.28 | 0.03 | -0.11 | 0.12 | 0.13 | 0.12 | 0.17 | -0.05 | -0.07 | 1.00 |  |  |  |  |  |
| 11. Distance to urban areas | 0.49 | -0.19 | -0.09 | -0.04 | -0.35 | 0.08 | -0.36 | 0.15 | 0.03 | -0.48 | 1.00 |  |  |  |  |
| 12. Density of paved roads | -0.40 | 0.22 | 0.14 | 0.11 | 0.30 | -0.17 | 0.20 | -0.14 | -0.10 | 0.13 | **-0.54** | 1.00 |  |  |  |
| 13. Distance to paved roads | **0.51** | -0.26 | -0.17 | -0.19 | -0.38 | 0.16 | -0.30 | 0.28 | -0.08 | -0.16 | **0.59** | **-0.82** | 1.00 |  |  |
| 14. Elevation | 0.31 | -0.16 | -0.06 | -0.12 | -0.16 | 0.17 | -0.31 | 0.05 | -0.15 | 0.11 | 0.18 | -0.17 | 0.28 | 1.00 |  |
| 15. Slope | 0.45 | -0.27 | -0.21 | 0.14 | -0.29 | 0.16 | -0.33 | -0.03 | 0.17 | -0.11 | 0.24 | -0.22 | 0.17 | 0.19 | 1.00 |
