# Supplementary material for: Using Species Distribution Models to Predict Potential Landscape Restoration Effects on Puma Conservation
Source: PLoS One. 2016 Jan 6;11(1):e0145232. doi: 10.1371/journal.pone.0145232 (PMC4703218; doi:10.1371/journal.pone.0145232)
Supplement: S4 Table — All variables were considered correlated to others (Pearson’s correlation ≥±0.5). (DOCX) [file pone.0145232.s007.docx]

Table S4. Environmental variables not used to develop the models for pumas in São Paulo State, Brazil. All variables were considered correlated to others (Pearson’s correlation ≥±0.5).

| Environmental variable | Description and source |
| --- | --- |
| Distance of native vegetation | Euclidean distance output raster measuring distance from every cell to the nearest native vegetation pixel in meters. |
| Distance of exotic forest crops | Euclidean distance output raster measuring distance from every cell to the nearest exotic forest crops pixel in meters. |
| Distance of protected areas | Euclidean distance output raster measuring distance from every cell to the nearest full protected area pixel in meters. |
| Distance of roads | Euclidean distance output raster measuring distance from every cell to the nearest state and federal highways pixel in meters |
| Distance of urban areas | Euclidean distance output raster measuring distance from every cell to the nearest urban area pixel in meters |
| Distance of watercourses | Euclidean distance output raster measuring distance from every cell to the nearest watercourse pixel in meters |
