# Supplementary material for: Using Species Distribution Models to Predict Potential Landscape Restoration Effects on Puma Conservation
Source: PLoS One. 2016 Jan 6;11(1):e0145232. doi: 10.1371/journal.pone.0145232 (PMC4703218; doi:10.1371/journal.pone.0145232)
Supplement: S5 Table — (DOCX) [file pone.0145232.s008.docx]

Table S4. Cross-tabulated areas calculated using ArcGIS 10.1 Spatial Analyst Zonal tool between the four probability of *Puma concolor* presence classes (i.e. low (values ≤ 0.17), medium (0.17 ≤ values ≤ 0.31), medium-high (0.31 ≤ values ≤ 0.50 and high (values > 0.50) habitat suitability and full protected areas (FPA), 10 km buffer zone from full protected areas (10KM), sustainable use areas (Áreas de Proteção Ambiental - APA) and Non-Protected Areas (NPA).

|  | | Puma’s habitat suitability area (km^2^) | | | | |
| --- | --- | --- | --- | --- | --- | --- |
| Land use | Low | | Medium | Medium-high | High |  |
| FPA | 2303.15 (3%) | | 1532.44 (2%) | 1834.62 (3%) | 3189.44 (24%) |  |
| 10KM | 10759.15 (16%) | | 9676.24 (8%) | 7220.86 (12%) | 4388.63 (33%) |  |
| APA | 6477.50 (10%) | | 7669.96 (7%) | 3802.13 (6%) | 932.30 (7%) |  |
| NPA | 45352.57 (71%) | | 93902.40 (83%) | 43881.05 (79%) | 4726.51 (36%) |  |
| Total in São Paulo | 64892.37 (100%) | | 112781.04 (100%) | 56738.66 (100%) | 13236.88 (100%) |  |
